# Combating a Global Threat to a Clonal Crop: Banana Black Sigatoka Pathogen *Pseudocercospora fijiensis* (Synonym *Mycosphaerella fijiensis*) Genomes Reveal Clues for Disease Control

**DOI:** 10.1371/journal.pgen.1005876

**Published:** 2016-08-11

**Authors:** Rafael E. Arango Isaza, Caucasella Diaz-Trujillo, Braham Dhillon, Andrea Aerts, Jean Carlier, Charles F. Crane, Tristan V. de Jong, Ineke de Vries, Robert Dietrich, Andrew D. Farmer, Claudia Fortes Fereira, Suzana Garcia, Mauricio Guzman, Richard C. Hamelin, Erika A. Lindquist, Rahim Mehrabi, Olman Quiros, Jeremy Schmutz, Harris Shapiro, Elizabeth Reynolds, Gabriel Scalliet, Manoel Souza, Ioannis Stergiopoulos, Theo A. J. Van der Lee, Pierre J. G. M. De Wit, Marie-Françoise Zapater, Lute-Harm Zwiers, Igor V. Grigoriev, Stephen B. Goodwin, Gert H. J. Kema

**Affiliations:** 1 Escuela de Biociencias, Universidad Nacional de Colombia, Medellín, Colombia; 2 Plant Biotechnology Unit, Corporación Para Investigaciones Biológicas, Medellín, Colombia; 3 Plant Research International, Wageningen University and Research, Wageningen, The Netherlands; 4 Department of Botany and Plant Pathology, Purdue University, West Lafayette, Indiana, United States of America; 5 US Department of Energy Joint Genome Institute, Walnut Creek, California, United States of America; 6 CIRAD, UMR BGPI, Montpellier, France; 7 USDA-Agricultural Research Service, West Lafayette, Indiana, United States of America; 8 Syngenta Biotechnology Inc., Research Triangle Park, United States of America; 9 National Center for Genome Resources, Santa Fe, United States of America; 10 Embrapa Mandioca e Fruticultura, Cruz das Almas, BA, Brazil; 11 University of Lavras, Lavras, Brazil; 12 National Banana Corporation of Costa Rica (CORBANA), La Rita de Pococí, Limón, Costa Rica; 13 Department of Forest and Conservation Sciences, The University of British Columbia, Vancouver, BC, Canada; 14 Natural Resources Canada, Canadian Forest Service, Laurentian Forestry Centre, Québec, QC, Canada; 15 Department of Genetics, Seed and Plant Improvement Institute, Karaj, Iran; 16 General Bioinformatics at Syngenta Crop protection Jeallots Hill International Research Centre, Bracknell Berkshire, United Kingdom; 17 Syngenta Crop Protection, Münchwilen AG, Stein, Switzerland; 18 Embrapa-LABEX Europe, Wageningen, The Netherlands; 19 University of California, Davis, Davis, California, United States of America; 20 Wageningen University, Laboratory of Phytopathology, Wageningen, The Netherlands; 21 CBS-KNAW Fungal Diversity Center, Utrecht, The Netherlands; Virginia Tech, UNITED STATES

## Abstract

Black Sigatoka or black leaf streak disease, caused by the Dothideomycete fungus *Pseudocercospora fijiensis* (previously: *Mycosphaerella fijiensis*), is the most significant foliar disease of banana worldwide. Due to the lack of effective host resistance, management of this disease requires frequent fungicide applications, which greatly increase the economic and environmental costs to produce banana. Weekly applications in most banana plantations lead to rapid evolution of fungicide-resistant strains within populations causing disease-control failures throughout the world. Given its extremely high economic importance, two strains of *P*. *fijiensis* were sequenced and assembled with the aid of a new genetic linkage map. The 74-Mb genome of *P*. *fijiensis* is massively expanded by LTR retrotransposons, making it the largest genome within the Dothideomycetes. Melting-curve assays suggest that the genomes of two closely related members of the Sigatoka disease complex, *P*. *eumusae* and *P*. *musae*, also are expanded. Electrophoretic karyotyping and analyses of molecular markers in *P*. *fijiensis* field populations showed chromosome-length polymorphisms and high genetic diversity. Genetic differentiation was also detected using neutral markers, suggesting strong selection with limited gene flow at the studied geographic scale. Frequencies of fungicide resistance in fungicide-treated plantations were much higher than those in untreated wild-type *P*. *fijiensis* populations. A homologue of the *Cladosporium fulvum Avr4* effector, *PfAvr4*, was identified in the *P*. *fijiensis* genome. Infiltration of the purified PfAVR4 protein into leaves of the resistant banana variety Calcutta 4 resulted in a hypersensitive-like response. This result suggests that Calcutta 4 could carry an unknown resistance gene recognizing PfAVR4. Besides adding to our understanding of the overall Dothideomycete genome structures, the *P*. *fijiensis* genome will aid in developing fungicide treatment schedules to combat this pathogen and in improving the efficiency of banana breeding programs.

## Author Summary

Black Sigatoka or black leaf streak disease, caused by the ascomycete fungus *Pseudocercospora fijiensis*, inflicts huge costs on banana producers, due to crop losses and expenses for disease control. The global banana export trade relies on Cavendish clones that are highly susceptible to *P*. *fijiensis*. Sustainable production of the world’s number one fruit requires a better understanding of host resistance and sophisticated management of fungicide resistance in the pathogen. In the *P*. *fijiensis* genome sequence we identified an effector that induced an HR-like necrosis on a resistant banana accession but not on a susceptible cultivar. If validated, this assay may be useful for identifying resistance in banana breeding programs. We also used the genomic sequence to develop highly polymorphic molecular markers for analyzing *P*. *fijiensis* field populations and identified a strong enrichment (nearly 100%) for fungicide resistance markers in fungicide-treated banana plantations compared to untreated wild-type populations. This rapid evolution of fungicide resistance poses an immediate threat to sustainable banana production.

## Introduction

Black Sigatoka or black leaf streak disease (BLSD), caused by the Dothideomycete fungus *Pseudocercospora fijiensis* (previously: *Mycosphaerella fijiensis*) [1], is a major threat to global banana production [2]. The disease is part of the Sigatoka complex, which involves two other closely related pathogens in addition to *P*. *fijiensis*: *P*. *musae* (previously: *M*. *musicola*) causal agent of yellow Sigatoka disease; and *P*. *eumusae* (previously: *M*. *eumusae*) causal agent of the eumusae leaf spot disease. Among the three species, *P*. *fijiensis* is the most aggressive and predominant member of the Sigatoka disease complex worldwide. These pathogens occur exclusively on the foliage of bananas and plantains, with continuous sexual and asexual reproduction in nature [1,3–5].

BLSD was first reported in the Sigatoka Valley of the Fiji islands during the 1960s and has since spread to nearly all banana-producing areas worldwide. It can only be managed by intensive fungicide applications, requiring weekly interventions throughout the year in most production areas. Black Sigatoka inflicts huge costs on global banana production, surpassing US $500 million per year [6]. Expenses for fungicide treatments usually represent more than 35% of total production costs [7,8]. Infection with *P*. *fijiensis* also results in crop losses and massive indirect costs by inducing early ripening of the fruit, making it unsuitable for sale with concomitant effects on the export trade and the retail sector.

Export banana cultivars are sterile, triploid plants that can only be propagated clonally and are grown in huge monocultures of genetically identical individuals. The international banana trade is based solely on a few closely related clones of the Cavendish type, all of which are highly susceptible [6]; disease management, therefore, relies primarily on fungicide applications with enormous environmental impacts [9]. Moreover, the selection pressure on *P*. *fijiensis* populations continuously reduces the efficacy of fungicides resulting in control failures and unmanageable levels of disease [2,7]. Therefore, there is an urgent need for scientific discoveries that will lead to the development of better methods for protecting banana crops, both for export fruit production and for small holders around the world who rely on bananas as a staple food [2].

Taxonomically, *Pseudocercospora* belongs to the order Capnodiales in the class Dothideomycetes, previously known as the *Loculoascomycetes* [10], which is the largest and most diverse class of ascomycete fungi comprising over 20,000 species. Dothideomycete fungi include endophytes and epiphytes of plants, but also saprobes degrading cellulose and other complex carbohydrates of dead plants, and plant pathogens [11]. The latter cause a range of diseases in various key food, fiber and fuel crops, including *Zymoseptoria tritici* (septoria tritici blotch of wheat) [12], *Venturia inaequalis* (apple scab) [13], and *Leptosphaeria maculans* (blackleg of Brassica crops) [14]. Therefore, genome sequences of several Dothideomycetes have been published [15–22] or are in the process of being completed (http://genome.jgi.doe.gov/dothideomycetes/dothideomycetes.info.html). The genome sequence of *Z*. *tritici* is the reference for all other Dothideomycetes as it is the only one that has been completely finished [21].

The poor experimental amenability of *P*. *fijiensis* has significantly hampered progress in understanding its basic biology [9] and the development of research tools. For instance, infection assays are cumbersome due to the need for very specific environmental conditions with respect to temperature, light and relative humidity, and the slow development of the disease that may take up to 50 days until symptoms are expressed [23,24]. Therefore, basic information on pathogenesis is not available and almost nothing is known about the genetic basis of disease resistance in banana germplasm [25,26]. Hence, new tools and research methods are needed to better understand the disease and ensure continued production of the world’s number one fruit, which is a staple food for millions of people in many developing countries.

A previous comparative analysis of 18 Dothideomycetes genomes [19] included that of *P*. *fijiensis* isolate CIRAD86 for a global analysis of genome organization and evolution. However, *P*. *fijiensis* was not the primary focus of that analysis and few specifics were discussed. Here we focus on the genome sequence of *P*. *fijiensis* isolate CIRAD86, describe the sequence of a second isolate, CIRAD139, and analyze in detail the species’ genome structure, content and function with a goal of delivering new data that could give clues for global disease management of this devastating banana pathogen.

## Results

### Sequencing, assembly and annotation of the *P*. *fijiensis* genome

The genomes of the *P*. *fijiensis* isolates were sequenced using either Sanger technology (CIRAD86) or Illumina for resequencing (CIRAD139a). The final assembly size of ~74 Mb consisted of 56 scaffolds with the largest at 11.8 Mb and an N50 of 50 Kb. Inclusion of a newly made genetic map facilitated assembly of the physical genome (S1 Table).

**Fig 1:**
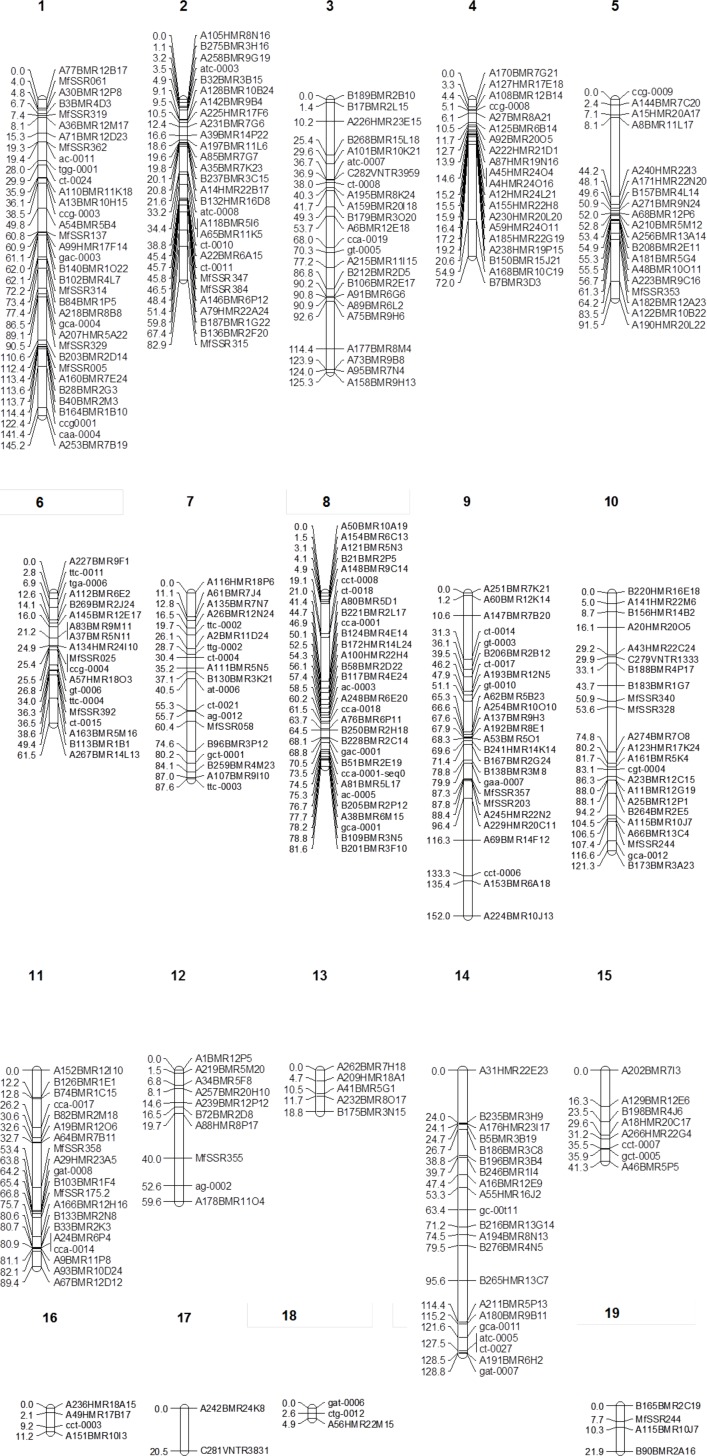
Genetic linkage map of *Pseudocercospora fijiensis* constructed from segregation data at 322 loci (233 DArT, 86 SSR and 3 minisatellite markers) among 135 individuals of a cross between the sequenced isolates CIRAD86 and CIRAD139A. The Diversity Arrays Technology (DArT) markers were named according to the output of proprietary DArT analysis software. For each of the 19 linkage groups (listed on top) the cumulative map distances (cM) as calculated using the Haldane mapping function are shown to the left.

Genetic map construction involved 376 loci that segregated in the progeny of the mapping population, among which 322 (233 DArT, 86 SSR, 3 minisatellite) markers were mapped into 19 linkage groups (Fig 1). The number of loci per linkage group varied from 2 to 35 with an average of 17 and linkage groups 1, 2, 8 and 9 contained the largest numbers of markers with 35, 29, 31 and 26, respectively. Map distances between consecutive markers varied from 0 to 20.4 cM with the largest gaps between markers on linkage groups 14 and 17, of 6.1 and 20.4 cM, respectively (Fig 1).

After filtering for EST support, completeness and similarity to other species, 13,107 genes were structurally and functionally annotated. The average gene length in the version 2 assembly is 1,833 nt with 3.62 exons per gene; 88%, are complete with start and stop codon, 74% have similarity support, and 49% have Pfam domains (S2 Table). Most of the gene models (96%) are located in 12 scaffolds, numbers 1–10, 12 and 19. Gene density in these 12 scaffolds varies from 151 to 229 per Mb, but gene density for the remaining scaffolds larger than 0.5 Mb drops to only 2 to 94 genes per Mb (S1 Table). More detailed information on the assembly, annotation and EST support data can be found in S1 Text.

### Genome structure

#### The *Pseudocercospora fijiensis* genome is greatly expanded

**Fig 2:**
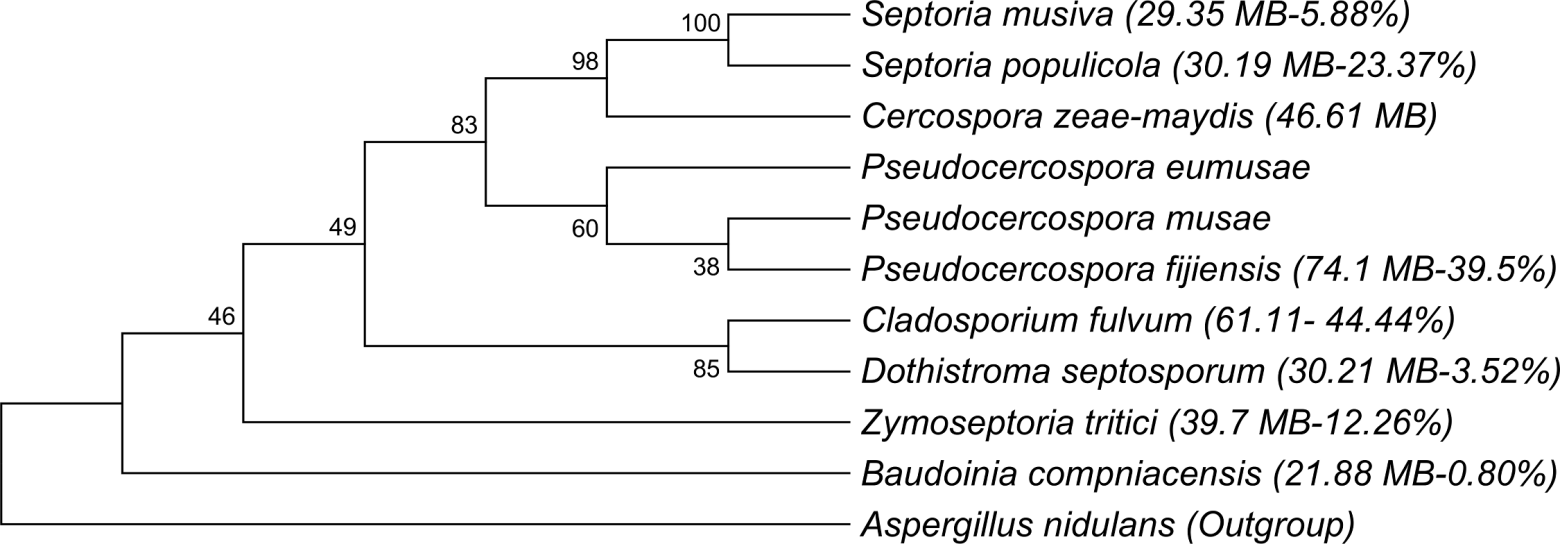
Phylogenetic analysis showing the placement of Dothideomycete species within the Capnodiales with expanded genomes. At least two genome expansions may have taken place; one leading to the banana pathogen *Pseudocercospora fijiensis* and one that contributed to its close relative the tomato pathogen *Cladosporium fulvum*. Genome sizes and percentages of the genome containing repeat elements are indicated in parentheses.

**Fig 3:**
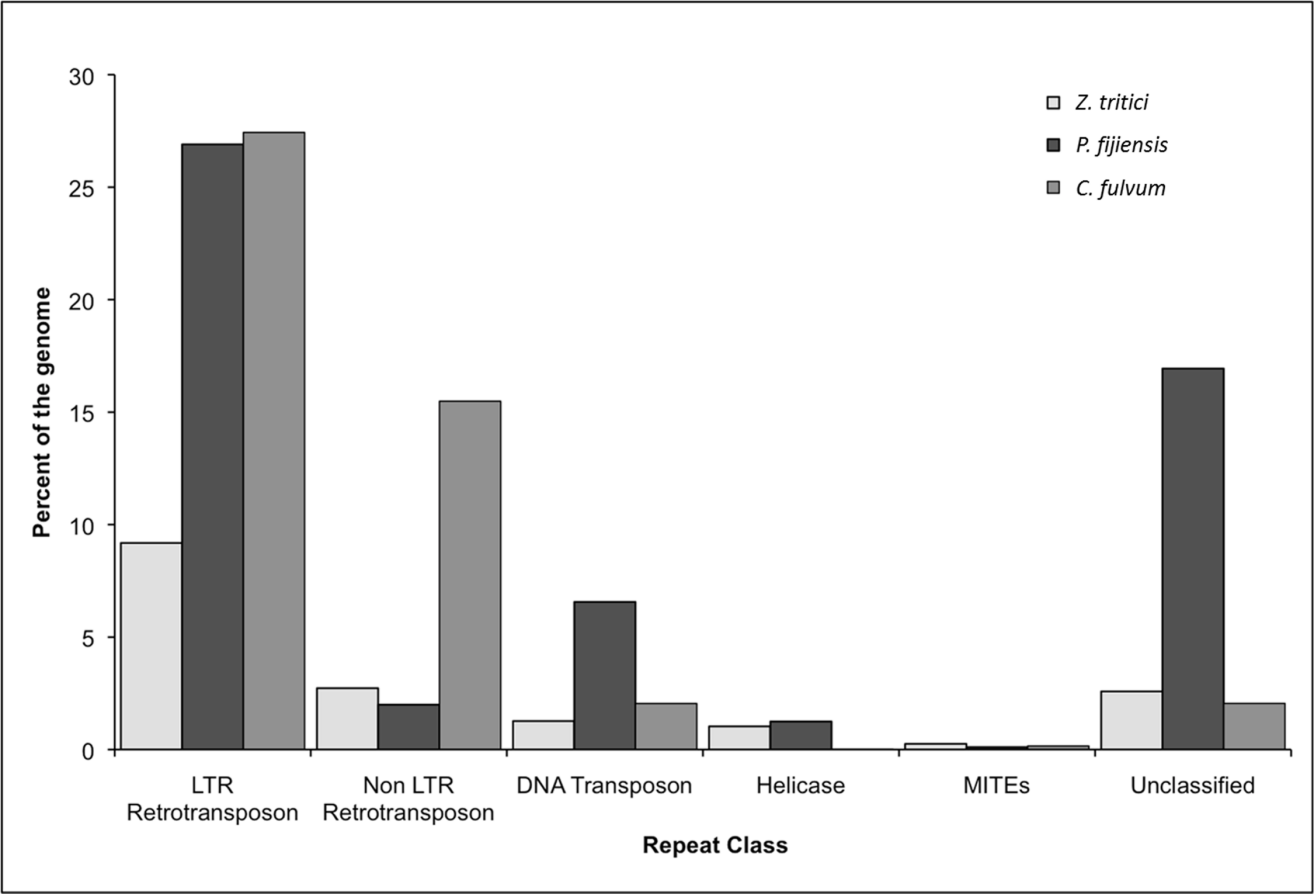
Comparison of repeat classes among *Zymoseptoria tritici*, the only Dothideomycete with a completely sequenced genome, *Pseudocercospora fijiensis* and *Cladosporium fulvum*, the only other Dothideomycete known to have a transposon-expanded genome.

The 74-megabase genome of *P*. *fijiensis* is greatly expanded relative to those of other related Capnodiales such as *Sphaerulina musiva* (previously *Septoria musiva* with teleomorph *Mycosphaerella populorum)*, *S*. *populicola* (previously *Septoria populicola* with teleomorph *Mycosphaerella populicola)* and less related species such as *Dothistroma septosporum*, *Baudoinia compniacensis*, and *Z*. *tritici*, but less so compared to *C*. *fulvum*, the closest Capnodiales relative sequenced and only other Dothideomycete with an expanded genome of 65 Mb (Fig 2). The predominant repetitive elements in the *P*. *fijiensis* genome belong to the long terminal repeat (LTR) retrotransposons (50%) (Fig 3), which is much higher than in *Z*. *tritici*, but similar to the proportion seen in *C*. *fulvum*. Compared to these other two species, the genome of *P*. *fijiensis* contained much higher percentages of repetitive DNA and unclassified transposons, whereas that of *C*. *fulvum* had the highest percentage of non-LTR retrotransposons among the three species (Fig 3). The estimated number of gene models is 13,107, which is approximately 28% and 34% higher than in *S*. *musiva* and *S*. *populicola*, respectively (Table 1) and 7% smaller than *C*. *fulvum*. Using the 80:80 criterion [27], i.e., 80% sequence identity across 80% alignment length, all of the *P*. *fijiensis* repeat families were unique. However, using a 70:70 cutoff criterion, elements from 50 *P*. *fijiensis* repeat families, amounting to 449 kb, were similar to those in the *C*. *fulvum* genome. A non-LTR repeat family from *P*. *fijiensis* (family 6), with an average element length of 4.9 kb, had the highest representation with 36 copies in the *C*. *fulvum* genome.

**Table 1:** Comparative genome statistics of the version 2 assembly of *Pseudocercospora fijiensis*, and several other sequenced fungi in the order Capnodiales.

Genome statistic	*P*. *fijiensis* V 2.0	*C*. *fulvum* V 1.0	*Z*. *tritici* V 2.0	*B*. *compniacensis* V1.0	*D*. *septosporum* V1.0	*S*. *populicola* V 1.0	*S*. *musiva* V 1.0
Genome size	74 MB	61.11 MB	40 MB	21.88 MB	30.21 MB	33.19 MB	29.35 MB
Scaffolds	56	4865	21	19	20	502	72
Scaffolds > 50 Kb	28	N.A*	21	17	14	141	13
Largest scaffold	11.8 MB	0.53 MB	6.0 MB	2.03 MB	5.1 MB	1.06 MB	5.11 MB
Percent in scaffolds > 50 KB	99.8	N.A	100	N.A	N.A	N.A	N.A
Gene models	13,107	14,127	10,952	10,513	12,580	9,739	10,233
Coverage	6.9×	N.A	8.9×	43x	34x	18x	35x

*N.A. Data not available at respective genome site.

**Fig 4:**
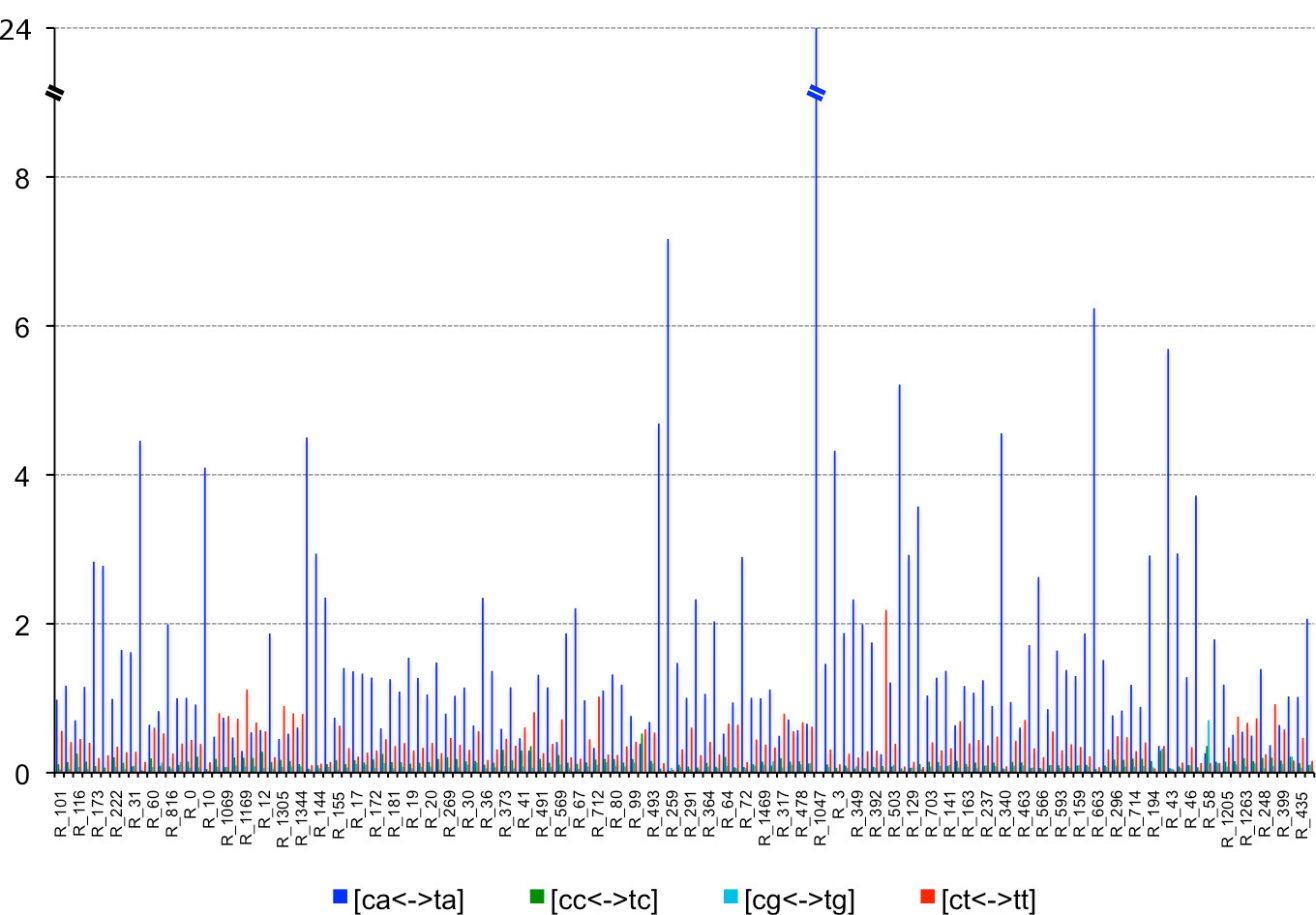
Repeat-induced point mutation (RIP) dinucleotide bias in *Pseudocercospora fijiensis* genome. A clear CA <-> TA dinucleotide bias is observed in *P*. *fijiensis* repetitive families, indicating that RIP likely occurs and mutates CA nucleotide pairs to CT.

Analysis of repeat-induced point mutation (RIP) showed a clear CA<-> TA dinucleotide bias in the repetitive elements identified in the *P*. *fijiensis* genome (Fig 4). Some families also showed a CT<->TT dinucleotide bias. A similar pattern has been observed in a number of ascomycete genomes, including *Parastagonospora nodorum* [28].

Repetitive elements often clustered to form blocks of AT-rich DNA. When an average DNA content of 45% or less was used to define AT-rich regions, a total of 1,865 AT blocks were identified in the *P*. *fijiensis* genome, ranging in length from 1 to 514 kb. These blocks account for 45 Mb (61%) of the *P*. *fijiensis* genome and 84% comprised repetitive sequences. A total of 482 (4%) genes were associated with the AT blocks. About 20% (96) of these genes have associated annotations and 6% (28) can potentially be secreted.

If a lower value of 40% average percent GC is used, the number of AT blocks diminished drastically to 640, amounting to a total length of 18 Mb or approximately 25% of the *P*. *fijiensis* genome. Repetitive sequences make up 84% and 152 (1.2%) genes were associated with these AT-rich blocks. Approximately 22% (33) of the genes associated with AT blocks have an annotation and about 10% (15) have signal peptides.

**Fig 5:**
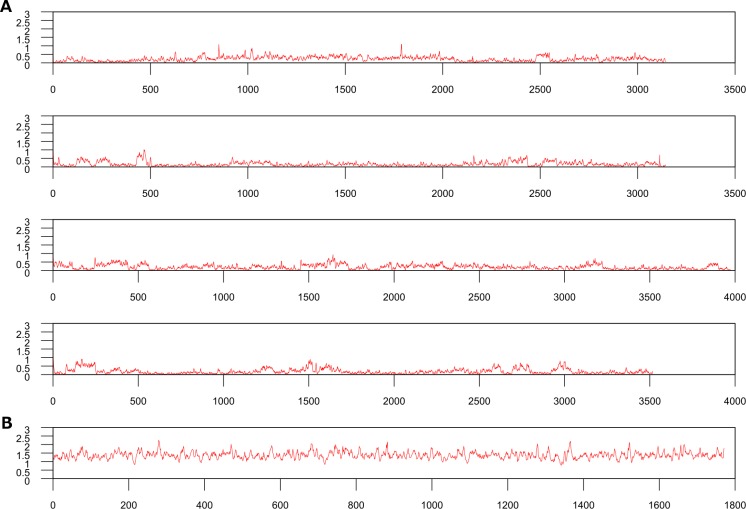
Comparison of the amount of repeat-induced point mutation (RIP) between AT-rich blocks and more GC-rich regions of the *Pseudocercospora fijiensis* genome as measured by the RIP index (CpA+TpG)/(ApC+GpT). (A) AT-rich blocks have a lower RIP index indicating a depletion of RIP-susceptible sites due to a higher frequency of RIP compared to (B) an AT-poor region (higher GC) of the genome, which has a higher RIP index reflecting very little RIP. Four AT-rich blocks are shown along with one AT-poor region for comparison. Length of each block in kilobases is shown along the x-axis and the RIP index (CpA+TpG)/(ApC+GpT) is shown on the y-axis.

The average RIP index was 0.2 in the AT-rich blocks as compared to a higher average RIP index of 1.37 across the rest of the genome. Plots of the RIP index were very low (indicating a high level of RIP) in the AT blocks (Fig 5A) but much higher (low RIP) in the regions of the genome with lower AT content (Fig 5B). As expected, there was a strong inverse relationship between GC content and the amount of RIP as measured by the index (S1 Fig). Very few of the genes (just over 3%) in AT-poor (= GC rich) regions of the genome showed any evidence of RIP (index of 1.0 or less) compared to a little over half (53%) of those in AT-rich regions (Table 2). In contrast, all but two out of 7,674 repeats in AT-rich regions showed evidence of RIP and almost 93% of those in AT-poor regions (Table 2). Exceptions were few and minor (S2 Fig).

**Table 2:** The repeat-induced point mutation (RIP) index calculated as (CpA+TpG)/(ApC+GpT) for genes^a^ and repeats^a^ in AT-poor and–rich regions of the *Pseudocercospora fijiensis* genome.

	Number of genes in		Number of repeats in	
RIP index	AT-poor blocks	AT-rich blocks	AT-poor blocks	AT-rich blocks
0.0	0	0	1	3
0.5	80	134	343	7,062
1.0	291	37	62	607
1.5	8,699	128	30	2
2.0	1,937	22	1	0
2.5	18	0	1	0
3.0	1	0	0	0
Total	11,026	321	438	7,674

^a^ The minimum sequence cutoff length for this analysis was 500 bp. A lower RIP index indicates a higher frequency of RIP mutations and vice versa.

**Fig 6:**
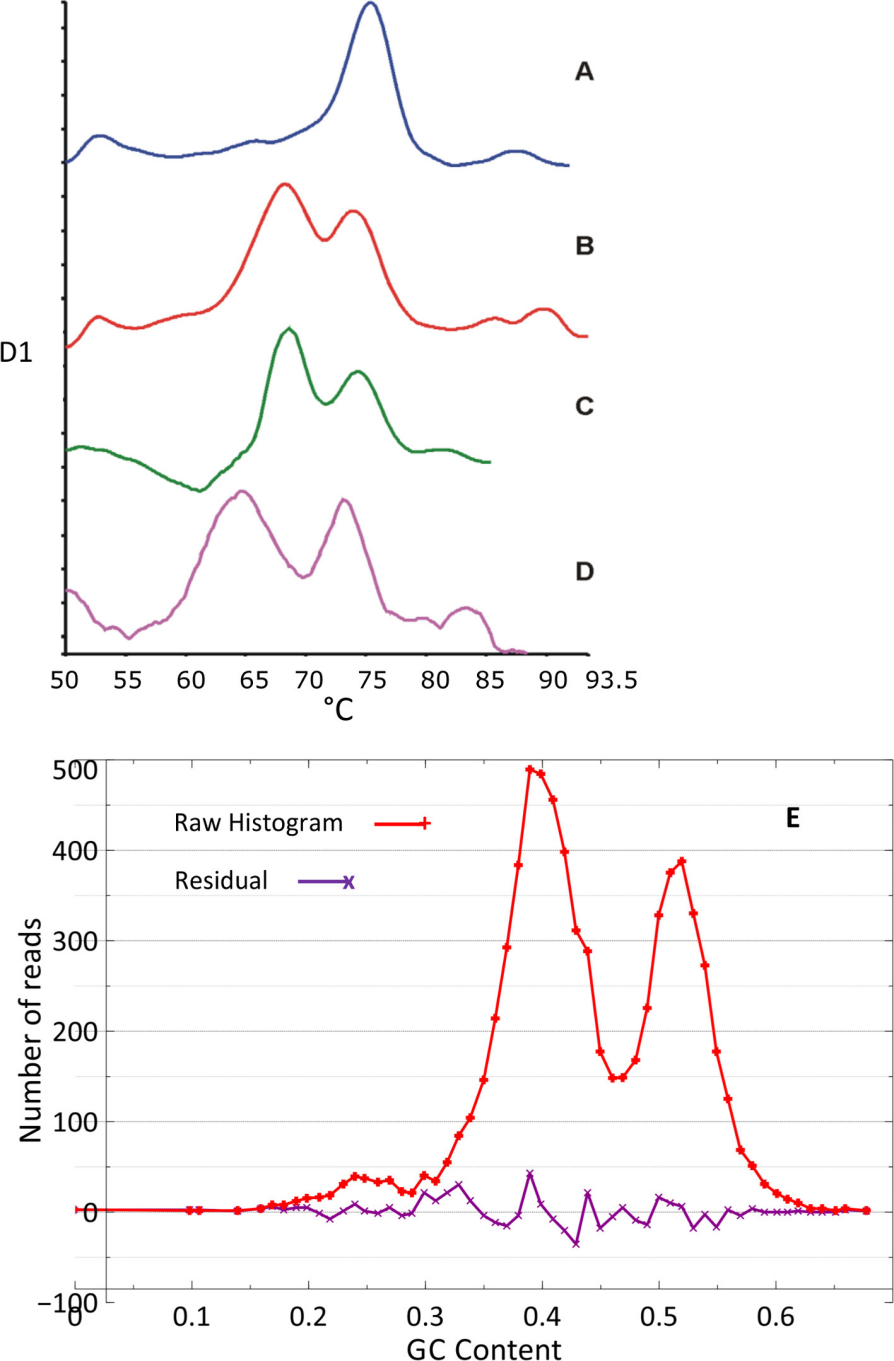
First-derivative graphs of melting curves of four different Dothideomycetes. Examples of first-derivative graphs of melting curves obtained for *Zymoseptoria tritici* (A), *Pseudocercospora fijiensis* (B), *P*. *eumusae* (C) and *P*. *musae* (D). E: A plot of G+C contents from sequence reads of *P*. *fijiensis*. This graph is very similar to the melting-curve analyses showing the difference in G+C content between the genomes of *P*. *fijiensis* and the other banana pathogens versus the *Z*. *tritici* genome.

First-derivative graphs obtained for melting profiles of *Z*. *tritici* showed a narrow curve with a single peak (Fig 6A); in contrast, those for *P*. *fijiensis* showed a broad curve with two peaks with G+C contents of 39.4 and 51.6% indicating heterogeneity (S3 Table). This agreed with the GC plots of sequence reads from *P*. *fijiensis*, which clearly showed a double-peak phenomenon, the lower peak corresponding to transposon-rich regions (Fig 6E).

The melting profiles obtained from the DNA of both *P*. *eumusae* and *P*. *musae* also demonstrated a double-peak pattern of genomic G+C content. The G+C content pattern in *P*. *eumusae* was almost identical to that in *P*. *fijiensis* with peaks at 39.6 and 51.6%. In *P*. *musae*, both peaks corresponded to lower, albeit still comparable, G+C contents of 37.2% and 50.9% (Fig 6A–6D, S3 Table). Plotting genome size on a phylogenetic tree of the Capnodiales identified at least two expansions, one leading to *P*. *fijiensis* and the second to the biotrophic tomato pathogen *C*. *fulvum* (Fig 2).

**Fig 7:**
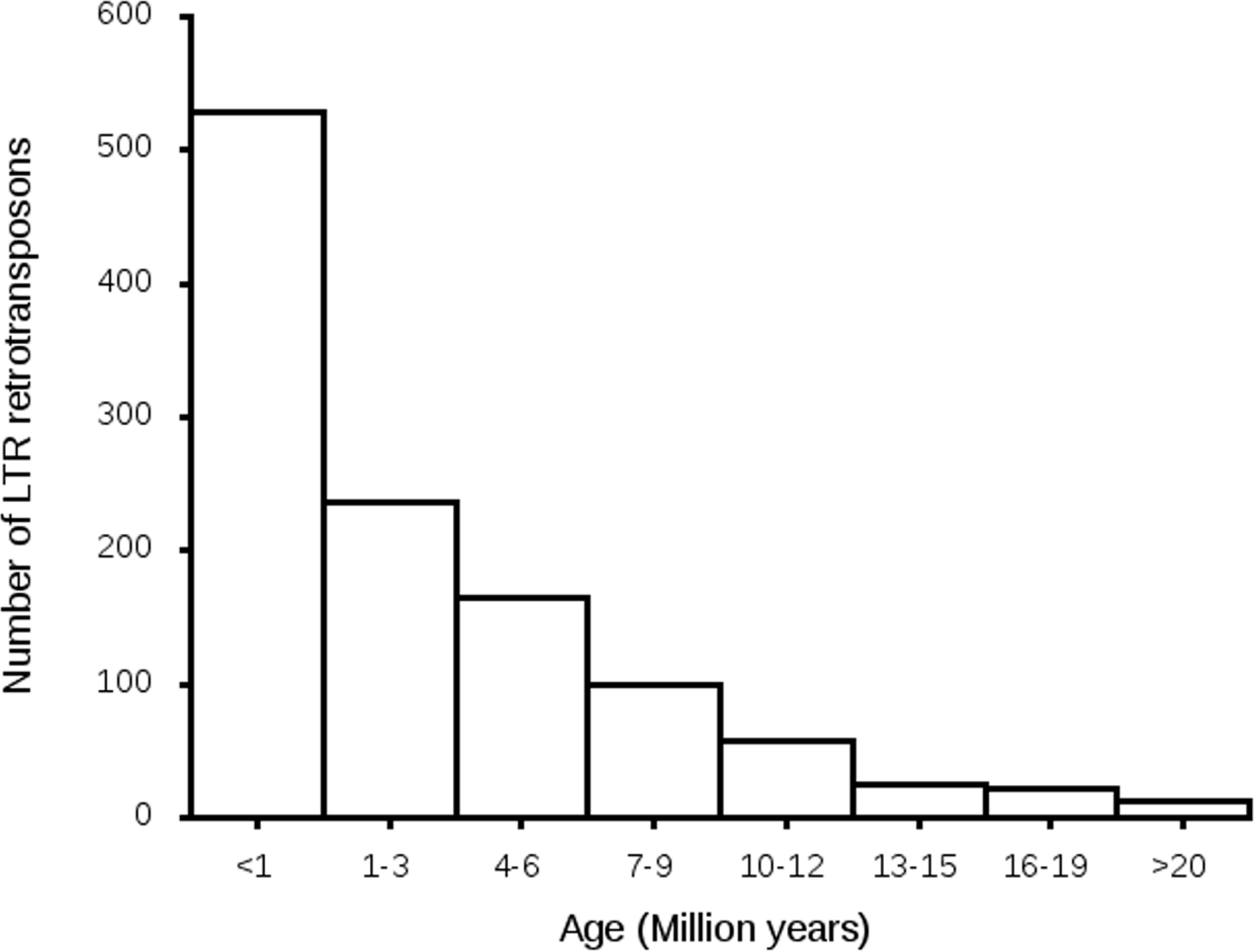
The numbers of long terminal repeat (LTR) retrotransposons in hypothetical age bins from less than one to more than 20 million years. Estimated age of each transposon was calculated using the number of differences between its left and right repeats. These are considered identical at the time of insertion so all changes are likely due to mutations that occurred after transposition. All transition mutations were excluded to minimize the effects of repeat-induced point mutation.

To estimate the age of the transposon expansion in the *P*. *fijiensis* genome, 1,147 bona fide, full-length LTR retrotransposons were used. Of these, 529 elements (46% of the total) had LTRs that were highly similar in terms of mutations accumulated over time with a hypothetical insertion age of less than one million years (Fig 7). Many older elements also were identified (Fig 7) but these decreased with time, indicating that most of the transposon insertions occurred relatively recently.

#### Electrophoretic karyotyping suggests variability in genome content and/or organization among isolates

**Fig 8:**
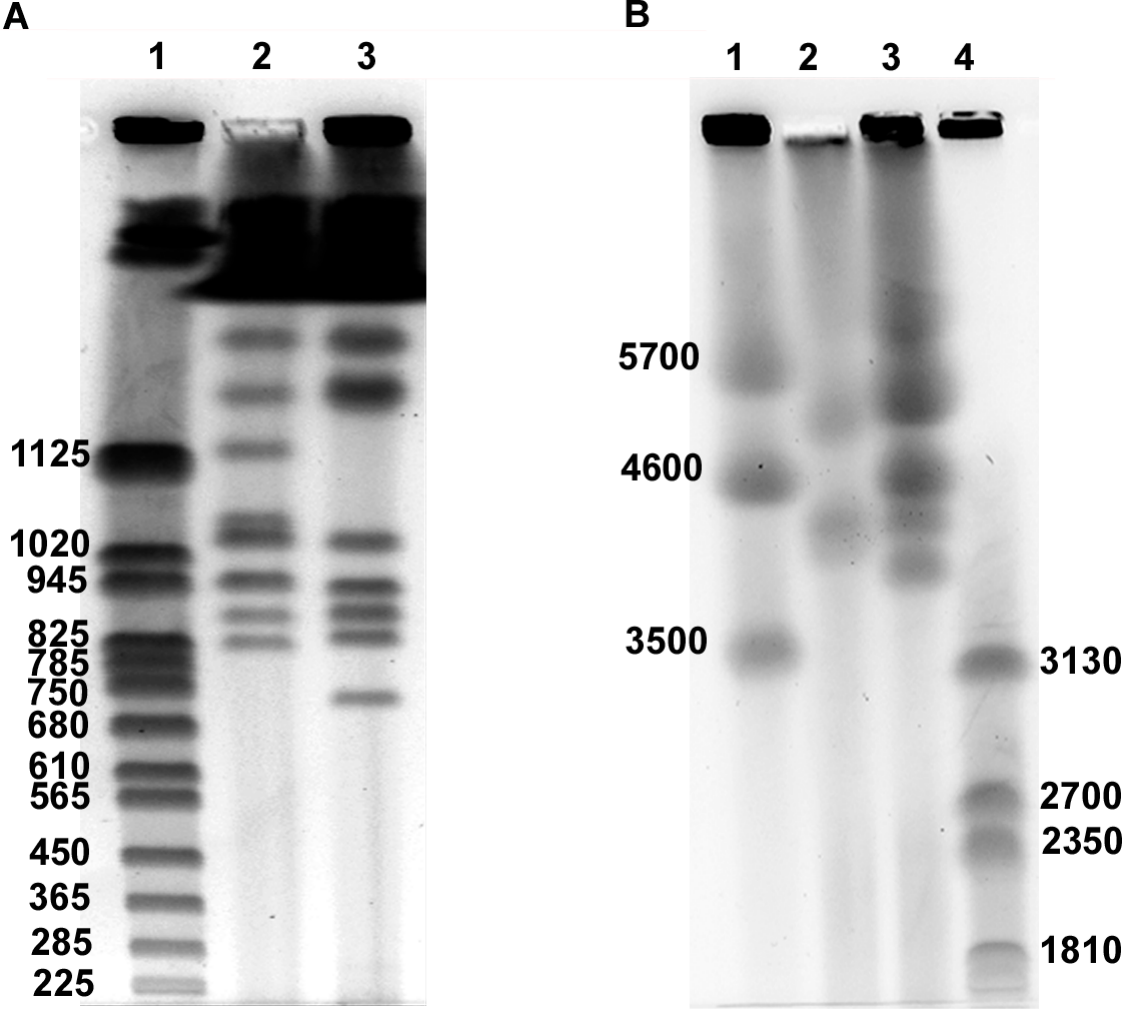
Electrophoretic karyotypes of two strains of *Pseudocercospora fijiensis*. A) Bands separated with conditions for small chromosomes. Lane 1, chromosomes from *Saccharomyces cerevisiae* as high-molecular-weight (HMW) marker; lane 2, strain CIRAD86; lane 3, strain E22. B) Bands separated under conditions to resolve medium and large chromosomes. Lane 1, chromosomes from *Schizosaccharomyces pombe* as HMW marker for large chromosomes; lane 2, strain CIRAD86; lane 3, strain E22; lane 4, chromosomes from *Hansenula wingei* as HMW marker for medium chromosomes in size. Marker sizes are in Kb.

Pulsed-field gel electrophoresis of the CIRAD86 and E22 strains showed small and large chromosomes, but no chromosomes in the medium range of 1.5 to 3.9 Mb. Isolate CIRAD86 showed 11 bands representing chromosomes, four of which appeared to be composed of double, co-migrating bands (Fig 8A). Small chromosomes were in the size range of 0.83 to 1.45 Mb. Bands of 0.95 and 1.03 Mb showed approximately twice the intensity and were assumed to represent at least two chromosomes each (Fig 8A). Conditions for separation of large chromosomes showed a band of 5.2 Mb, a co-migrating chromosomal band of 4.33 and a smaller band of 4.27 Mb (Fig 8B). Strain E22 showed at least 12 bands in total, five of which likely contain co-migrating chromosomes. Small chromosomes were in the range of 0.70–1.45 Mb, and large chromosomes had estimated lengths between 4.05 and 6.80 Mb (Fig 8B). Additionally a comparison of small chromosomal bands of different strains originating from a single banana field showed that every isolate contained between five and nine small chromosomal bands with unique length polymorphisms (S3 Fig), indicating substantial variation in genome content and/or genome organization among individuals.

#### Synteny analysis suggests a core set of 12 chromosomes

**Fig 9:**
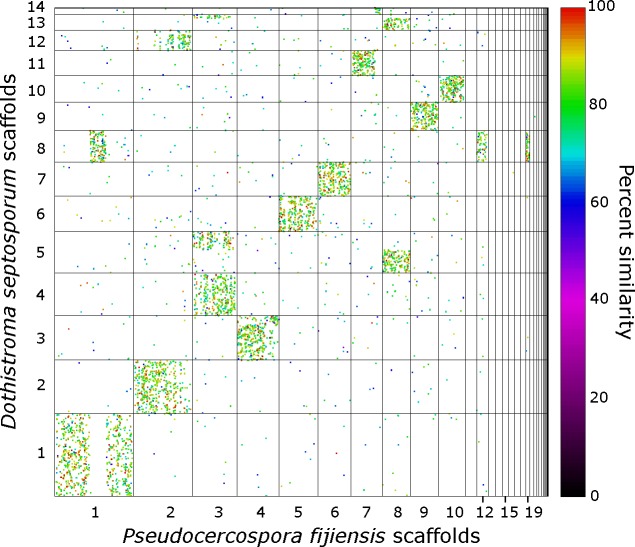
Dot plot showing mesosynteny between the scaffolds of *Pseudocercospora fijiensis* and *Dothistroma septosporum*.

Analysis of similarity between the genomes of different Dothideomycetes shows a high degree of conservation of genes in syntenic scaffolds. Mesosynteny was observed between *P*. *fijiensis* and all other Dothideomycetes analyzed, including the Capnodiales *B*. *compniacensis*, *Cercospora zeae-maydis*, *C*. *fulvum*, *D*. *septosporum* (Fig 9), *S*. *musiva* (S4 Fig), *Zasmidium cellare* and *Z*. *tritici*, the Pleosporales *Cochliobolus heterostrophus*, *L*. *maculans*, *Pyrenophora tritici-repentis* and *P*. *nodorum*, and the Hysteriales species *Hysterium pulicare*. Microsyntenic blocks of up to 10 Kb were found only with the closest relatives *C*. *fulvum*, *D*. *septosporum* and *Z*. *tritici* (*S*. *musiva* was not tested for this analysis). No macrosynteny was observed between *P*. *fijiensis* and any of the presently sequenced Dothideomycetes.

**Fig 10:**
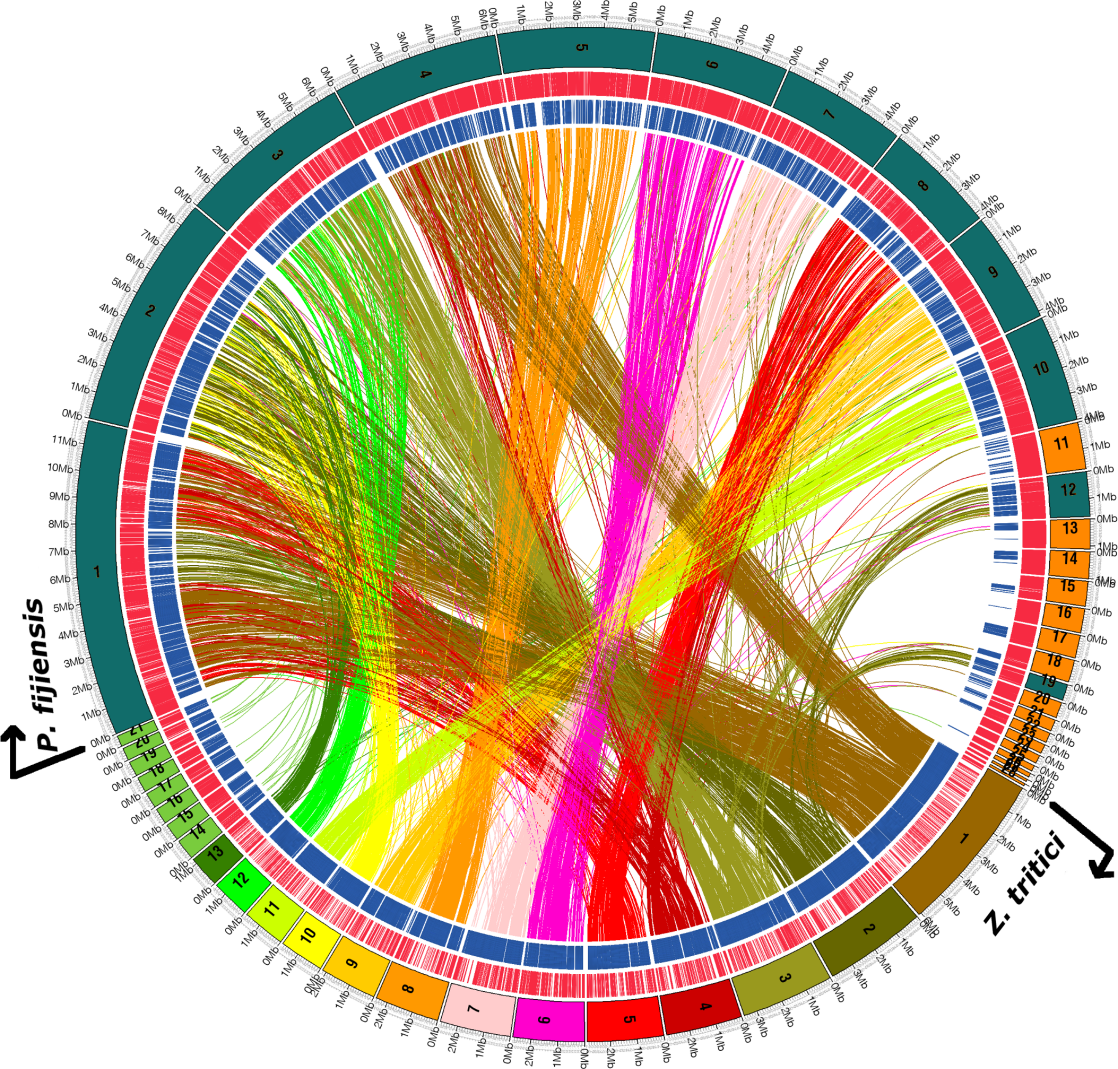
Genome-wide nucleotide comparison between *Zymoseptoria tritici* (lower half of the circle) and *Pseudocercospora fijiensis* (upper half of the circle). The longest 28 scaffolds from *P*. *fijiensis* are shown. Gene content is conserved but is scattered among different chromosomes between these two fungi. There were no significant hits to dispensable chromosomes of *Z*. *tritici* (14–21). The 12 major scaffolds of *P*. *fijiensis* showing synteny are labeled in dark blue-green and the other 16 scaffolds are labeled in orange.

Using *Z*. *tritici* as a reference it is clear that gene content is conserved among large blocks of chromosomes. For example, scaffold 1 of *P*. *fijiensis* shows synteny with chromosomes 1, 4 and 5 of *Z*. *tritici*, scaffold 2 with chromosomes 2, 10 and 13, whereas scaffold 6 of *P*. *fijiensis* shows synteny only with *Z*. *tritici* chromosome 6 (S5 Fig). Interestingly, no significant synteny was found between any of the scaffolds of *P*. *fijiensis* and the dispensable chromosomes of *Z*. *tritici* (Fig 10), supporting the hypothesis of their independent origin, possibly by recent horizontal transfer, in the latter species [17].

Most of the synteny found in *P*. *fijiensis* with *Z*. *tritici* as well as with all other Dothideomycetes tested is present in scaffolds 1 through 10, 12 and 19. In addition, these scaffolds showed the highest percent of genes with expression data (0.8% or greater), all of which suggest that these 12 scaffolds might represent the core genome. Many of the smaller scaffolds in the *P*. *fijiensis* genome have the physical characteristics observed for dispensable chromosomes in *Z*. *tritici*; they are smaller, with lower G+C contents and gene densities (S1 Table). Based on these criteria, the core genome of *P*. *fijiensis* comprises 63.9 Mb or almost 87% of the genome, while the remaining 13% may be a dispensome.

Analysis of the synteny plots also showed some past chromosomal rearrangements. For example, approximately 22% of the gene content of the central part of scaffold 1 of *P*. *fijiensis* was missing from the largest scaffolds of *D*. *septosporum* (Fig 9) and *S*. *musiva* (S4 Fig), and instead was found on scaffolds 8 and 7 of those species, respectively. This difference also was seen in the comparison with the more distantly related *Z*. *tritici* (S5 Fig), although the result was not as clear and more chromosomes were involved. In a direct comparison, scaffold 1 of *D*. *septosporum* showed complete mesosynteny with scaffold 1 of *S*. *musiva* (S6 Fig), suggesting that the central part of *P*. *fijiensis* scaffold 1 might have translocated after the divergence of all three species from an unknown common ancestor. The chromosome that likely supplied the translocation, corresponding to scaffolds 8 and 7 in *D*. *septosporum* and *S*. *musiva*, respectively, showed 1:1 mesosynteny in the direct comparison between those two species (S6 Fig), but also showed mesosynteny with *P*. *fijiensis* scaffolds 12 and 19 (Figs 9 and S4). This suggests that scaffolds 12 and 19 of *P*. *fijiensis* may belong to a single chromosome that has not been assembled completely. Similar analyses identified a possible translocation or incomplete assembly involving scaffolds 3 and 8 of *P*. *fijiensis*, which correspond to scaffolds 5 (Fig 9) or 4 (S4 Fig) of *D*. *septosporum* and *S*. *musiva*, respectively.

#### Re-sequencing of P. fijiensis isolate CIRAD139A shows a 12% difference in genome content

Among the more than 73 million reads of paired-end sequence data obtained for isolate CIRAD139A, 60% could be aligned uniquely to the *P*. *fijiensis* reference genome of isolate CIRAD86 (S4 Table). Another 28% of the reads aligned to multiple locations in the reference sequence, most likely due to duplications or repetitive elements in the genome. Almost 12% of the reads did not map to the reference, suggesting that some genome content present in CIRAD139A could be absent in the reference strain.

The numbers of polymorphisms varied widely among scaffolds roughly in proportion to size, and the number of SNPs was much higher than for indels on all scaffolds analyzed (S5 Table). Mean SNP frequency on each scaffold calculated across a 10-kb window was more uniform, ranging from 59.2 for scaffold 13 to 84.1 for scaffold 11 (S5 Table). Plotting the SNP density relative to gene density for the 21 largest scaffolds containing 99% of all gene models separated the scaffolds into two groups. One group contained most of the largest scaffolds and showed lower variability in SNP counts, while the second contained scaffolds with low gene densities and showed much more variability in SNP counts.

### Genome content

#### Decreased numbers of pathogenicity-related genes

Cell wall degrading enzymes (CWDEs) that break down physical barriers, including cutin, are important pathogenicity factors, particularly in necrotrophic fungi. Comparison of the number of genes related to cell wall degradation in *P*. *fijiensis* with those in other fungi revealed a significant reduction, particularly when compared to necrotrophic Dothideomycetes. Cutinases, xylanases and chitinases are reduced three to five fold when compared to three fungi in the Pleosporales: *P*. *nodorum*, *P*. *tritici-repentis* and *C*. *heterostrophus* (Table 3). Additionally, carbohydrate-binding proteins, including those with chitin-binding and cellulose-binding modules as well as β glucosidases also are reduced (Table 3). EST support was found for four chitinases, 18 glucosidases, one cellulose binding and four chitin binding genes.

**Table 3:** Comparison of selected gene families with potential roles in pathogenicity among five Dothideomycete fungi and the saprotrophic Sordariomycete *Neurospora crassa*.

	*Pseudocercospora fijiensis*	*Zymoseptoria tritici*	*Parastagonospora nodorum*	*Pyrenophora tritici-repentis*	*Cochliobolus heterostrophus*	*Neurospora crassa*
Peptidases	189	187	381	265	248	168
Cutinases	7	6	0	8	13	0
Beta Glucosidase activity	9	2	6	13	13	3
Chitinases	5	3	36	17	21	8
Chitin binding	5	2	35	13	16	3
Cellulose binding	4	0	18	46	55	26
Xylanases	7	5	31	21	31	5
NRPS	13	11	18	13	11	5
Polyketide synthases	11	12	21	28	21	4
Map kinases	5	5	12	5	4	4
Peroxidases	29	22	32	26	28	10
Carbohydrate Metabolic process	127	77	231	204	209	129
O-glycosyl hydrolase activity	77	51	129	97	106	77

Similar to the CWDEs, the *P*. *fijiensis* genome shows a relatively low number of genes involved in the production of secondary metabolites, such as polyketide synthases (PKSs), with approximately half the number of genes found in the necrotrophs *P*. *nodorum*, *P*. *tritici-repentis* and *C*. *heterostrophus*. Contrary to the situation for PKSs, genes encoding non-ribosomal peptide synthetases (NRPSs) are not reduced in *P*. *fijiensis*. Its genome encodes 13 NRPSs and one hybrid NRPS-PKS, which is comparable to the numbers found in other Dothideomycetes (Table 3). However, EST support was found for only six of the PKS genes and four of the NRPS and the hybrid NRPS-PKS genes. This low level of EST support might be a sampling phenomenon due to the EST coverage; none of the libraries came from *in planta* conditions where these genes are more likely to be expressed.

#### The *P*. *fijiensis* secretome

Filamentous fungal pathogens are able to modulate resistance responses in the plant cell by secreting a class of proteins known as effectors. In many fungal pathosystems, effectors are important pathogenicity or virulence factors that determine the success of a fungal infection [29,30]. The majority of described fungal effectors share many characteristics and belong to the class of small, secreted, cysteine-rich proteins (SSPs) [29]. A search of the genome with the above criteria showed that *P*. *fijiensis* possesses 172 genes encoding SSPs (smaller than 300 AAs in size) with four or more cysteine residues. Sixty-two percent of the *P*. *fijiensis* SSPs have no blast hits (107 proteins), 21% (37 proteins) have assigned GO terms and 23% have InterPro IDs other than SignalP (40 proteins). Thus, the number of potential SSP-encoding genes in *P*. *fijiensis* is 31% and 8% lower than in the genomes of *P*. *nodorum* (250 genes) and *Z*. *tritici* (187 genes), respectively (S7 Fig). These results accord with Ohm et al. [19] who found reduced numbers of SSPs in several Capnodiales.

Among the identified SSPs, one shows high similarity to *C*. *fulvum Avr4*, which is known to have a chitin-binding domain and is a well studied effector in the *C*. *fulvum*-tomato interaction [31]. This *P*. *fijiensis* putative *Avr4* (*PfAvr4*) homolog is a 121 amino acid protein present on scaffold 4 from co-ordinates 183261–183623 and was shown to protect *Trichoderma viride* cell walls against hydrolysis by plant chitinases through chitin binding and to trigger a *Cf4*-mediated hypersensitive response (HR) in tomato [32,33]. Additionally, three homologs of *C*. *fulvum* effector *Ecp2* were found, one of which was able to induce different levels of necrosis or HR in tomato lines depending on whether they lack or contain a putative corresponding Cf-ECP2 protein [32,33]. It seems highly likely that at least some of these *P*. *fijiensis* effector proteins that are similar to known effectors in *C*. *fulvum* will play a role in pathogenicity or virulence of *P*. *fijiensis* on banana.

### Genome function

#### Functional analysis of a putative effector protein

**Fig 11:**
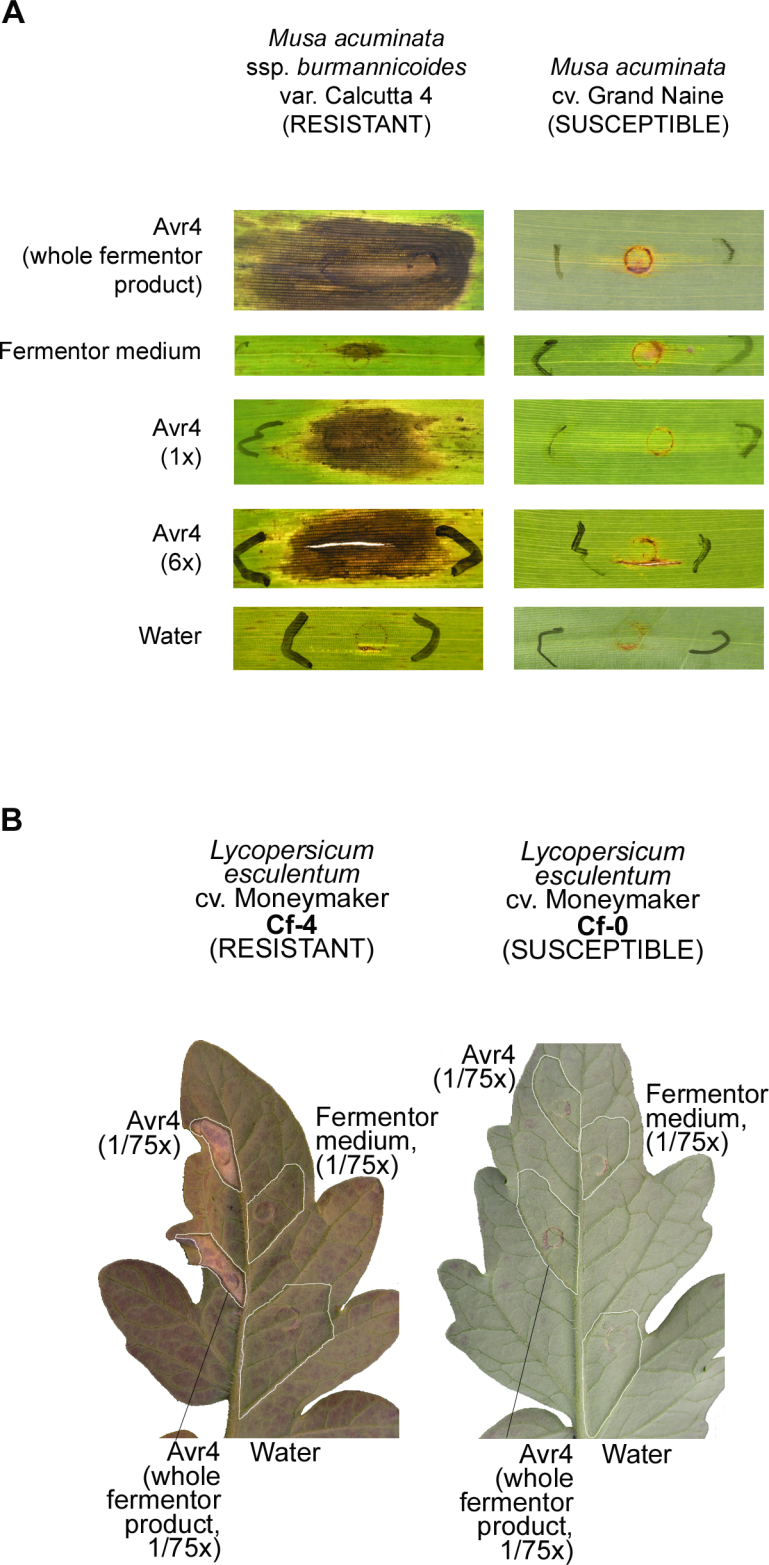
Infiltration of purified protein of the putative effector gene *PfAvr4* from *Pseudocercospora fijiensis* into leaves of banana and tomato. A: Infiltrations into leaves of resistant and susceptible banana varieties. B: Infiltrations into leaves of tomato with or without the *Cf4* resistance gene known to interact with PfAVR4. Experiments were done with crude fermentor product and concentrated or diluted product. Fermentor medium alone and water were used as controls.

Infiltrations into banana and tomato leaves were performed to test the hypothesis that *PfAvr4* acts as an avirulence factor in banana. Two different varieties were infiltrated with different concentrations of PfAVR4. Physical damage (small tear and occasional slight necrosis limited to the site of infiltration) caused by either the syringe or the fermentor medium was similar among varieties (Fig 11) and was less intense in tomato plants (Fig 11B). Both large and small plants of *M*. *acuminata* var. Grand Naine showed only physical damage with an occasional slight chlorotic effect at the infiltrated area (Fig 11A) after infiltrating the PfAVR4 protein; no hypersensitive response (HR) was observed at 10 days post infiltration (dpi) regardless of the protein concentration used.

In contrast, PfAVR4 triggered a clear HR-like necrosis when infiltrated into leaves of *M*. *acuminata* ssp. *burmannicoides* var. Calcutta 4, which has resistance against *P*. *fijiensis* (Fig 11A). The necrosis was already visible on large plants at 4 dpi, and was stronger by 10 dpi at both concentrations of PfAVR4. In small plants the earliest necrosis was observed at 10 dpi. Fermentor medium triggered a slight necrosis on both small and large plants of var. Calcutta 4, but this was very different from the HR-like necrosis induced by PfAVR4 and the combined effect triggered by the fermentor product (Fig 11A). Furthermore, tomato plants without a resistance gene (*Cf0* plants) showed only physical damage following infiltration, while those containing the *Cf4* resistance gene showed a HR to crude fermentor products containing PfAVR4 and to the purified PfAVR4 protein (Fig 11B).

#### Analyses of fungicide resistance and molecular markers within populations

In total, 621 hierarchically sampled *P*. *fijiensis* isolates were genotyped and partially phenotyped and showed that the commercial (sprayed) plantations were entirely or nearly fixed for quinone outside inhibitor (QoI) or strobilurin resistance (92–100%), whereas all 87 isolates sampled from the wild type, unsprayed San Carlos population were sensitive (S6 Table). Subsequently, we used the genome sequence to develop primers for five Variable Number of Tandem Repeat (VNTR) loci enabling population diversity analyses that were combined with assessment of the mating type loci. We observed that the ratios between the two mating type alleles *mat1-1* and *mat1-2* are not significantly different from 1:1 in each individual population as well as the overall total number of isolates (S6 Table), and that the VNTR loci in all populations are in gametic equilibrium and hence, could be used to estimate genetic differentiation between populations, which was small but statistically significant (S7 Table).

## Discussion

The genome of *P*. *fijiensis* is the largest among all of the Dothideomycetes sequenced to date; it is 3.4 times larger than that of the Dothideomycete with the smallest genome, *Baudoinia compniacensis*, 1.85 times larger than that of *Z*. *tritici* and 1.2 times larger than that of *C*. *fulvum*, which is related to *P*. *fijiensis* and also has an expanded genome [18]. Almost all of the increased size is due to the proliferation of LTR retrotransposons, as described here and in another publication [34]. The thermal denaturation results indicate that G+C content heterogeneity is not limited to *P*. *fijiensis*, but also occurs in its close relatives within a monophyletic clade of banana pathogens. Based upon the observed similarity of DNA composition between these three banana pathogens, we predict that the genomes of both *P*. *eumusae* and *P*. *musae* also are expanded and that all three pathogens that often co-occur in nature seem to have a recent common ancestor [4].

The only other member of the Capnodiales known to have a similarly expanded genome is the biotrophic tomato pathogen, *C*. *fulvum* [18]. Based on the positions of the species with expanded genomes on the phylogenetic tree (Fig 2), there appear to have been at least two independent expansions in genome size within the Capnodiales, one involving *P*. *fijiensis* (and most likely related banana pathogens) and another for *C*. *fulvum*. Lack of similarity between the transposable elements in the genomes of *P*. *fijiensis* and *C*. *fulvum* supports the hypothesis of independent expansions in each genome.

The terminal repeats of LTR retrotransposons are identical at the time of insertion and this provides a means to estimate the relative ages of transposable element insertions. This phenomenon has been very useful for estimating transposon insertion times in plants [35,36] but is less useful for fungi where RIP greatly increases the rate of mutations in repetitive sequences. Using this approach in the basidiomycete *Laccaria bicolor*, three periods of transposon insertion were identified, ranging in age from 0 to 59 million years ago (Mya) [37]. Whether RIP occurs in *L*. *bicolor* is not known, but its genome does not contain the genes known to be required for RIP in other fungi [38] and no evidence for RIP was found among transposons in the genomes of other members of the Agaricomycotina [39] so it seems unlikely. Therefore, this approach for estimating the ages of transposon insertions is most likely valid for *L*. *bicolor*.

A similar analysis of LTRs of transposons in the genome of *Z*. *tritici* yielded hypothetical insertion ages ranging from 0 to 5.7 Mya, with the strong caveat that the times were probably vastly overestimated due to RIP [40]. Even with the bias introduced by RIP, the estimated ages of transposon insertions in the genome of *Z*. *tritici* were an order of magnitude younger than those in *L*. *bicolor*, indicating that they must have occurred relatively recently. A more accurate approach to estimating transposon age in the presence of RIP would be to exclude the RIP-susceptible sites from analysis [41]. Using this approach, transposon insertions in *L*. *maculans* and closely related species in the Pleosporales, another large order of the Dothideomycetes, mostly were relatively recent, within the past four million years [41, 42]. For *P*. *fijiensis*, the results of a similar analysis clearly suggest a recent, rapid burst of LTR retrotransposon insertions. The young age and high proliferation rate of around 46% of the LTR retrotransposons suggest that *P*. *fijiensis* has a highly dynamic genome. Such a recent, high level of activity of retrotransposons can have evolutionary as well as regulatory implications for gene expression that can be better understood using genomic comparisons with other closely related species.

Large genome expansions due to amplifications of repetitive elements have been observed in other plant-pathogenic fungi. The published genome sequences and analyses of the powdery mildew fungi *Blumeria graminis* f. sp. *hordei*, *Erysiphe pisi* and *Golovinomyces orontii* show a marked genome expansion with a massive proliferation of non–LTR retrotransposons and a corresponding decrease in gene content [43]. The missing genes in these obligate biotrophs include enzymes for primary and secondary metabolism, carbohydrate-active enzymes, transporters, and secreted proteins such as effectors. The genome of *P*. *fijiensis* also shows a moderate decrease in certain gene families associated with pathogenicity such as PKSs and CWDEs when compared to necrotrophic Dothideomycetes such as *P*. *nodorum* or *P*. *tritici-repentis* [16]. In a similar way, the hemibiotrophs *Z*. *tritici* [17], *S*. *populorum* and *S*. *populicola* [19,22] also show a marked decrease in CWDEs [19], although not to the extent seen in the powdery mildews. This reduction in *Z*. *tritici* is thought to have evolved as a mechanism to evade detection by host defenses during stealth pathogenesis [17]. This hypothesis also could fit the lifestyle of *P*. *fijiensis* since it has an even longer biotrophic phase of up to 28 days before necrotic symptoms start to appear so may have a greater need for stealth [23]. However, the association is not perfect because the reduction in CWDEs in *Z*. *tritici* is greater, particularly for β glucosidases.

Thus, within Ascomycetes there seems to be a correlation between pathogenic lifestyle (biotrophic vs. hemibiotrophic vs. necrotrophic) and diversity of certain gene families such as PKSs and CWDEs. This correlation does not hold up when extended to other fungal groups. For example, rust fungi are obligate biotrophs with greatly expanded genomes due to either the proliferation of LTR retrotransposons similar to *P*. *fijiensis* for the wheat stem rust pathogen *Puccinia graminis*, or to class II DNA transposons for the poplar rust fungus *Melampsora larici-populina* [44]. However, in both rusts gene numbers were greatly expanded including those for SSPs that may be involved in interactions with their hosts [44]. Thus, evolution of a biotrophic lifestyle has involved very different changes in the genetic architecture of fungal genomes, from the vast reductions in gene content noted in the powdery mildew fungi to huge expansions for the rusts. Hemibiotrophs such as *P*. *fijiensis* fall in the middle of the continuum. The one constant is the increased genome size due to transposons, which seems much more common in biotrophic and hemobiotrophic pathogens compared to necrotrophs or saprotrophs.

The mechanisms of these transposon expansions remain mostly unknown. The two main unanswered questions revolve around the source of the invading elements and the mechanism by which they proliferate. The most obvious source would be their host plants, but so far there appears to be little evidence that transposons are being transferred from hosts to their pathogens. Biotrophic fungi should be the most suited for acquiring transposons because they are restricted to growing in a very limited ecological niche and have specialized feeding structures to retrieve nutrients from their hosts. For *P*. *fijiensis*, a search of the banana genome sequence revealed that transposable elements account for almost half of the *Musa* sequence with LTR retrotransposons representing the largest part [45,46], so the transposons might have come from banana. If not from the hosts, then they most likely have been acquired from other fungi or pests that are associated with the hosts. Horizontal transfer of genes has been shown in other fungi such as *P*. *nodorum* [47] and *Fusarium oxysporum* f. sp. *lycopersici* [48] and it could occur for transposons. Horizontal transfer has the potential to broaden host range and pathogenicity of fungal pathogens or even create a new pathogen from a non/pathogenic strain [47,48]. Solving the mystery about the origin of invading transposons is important for understanding the dynamics of fungal genome expansions, and the causal agents of the Sigatoka complex on bananas represent a good model to address such a question.

A little more is known about the mechanisms for transposon expansion after they have been acquired. Almost all fungi are capable of Repeat-Induced Point (RIP) mutation, a mechanism for identifying and mutating repetitive sequences [39,41,49–52]. For transposons, the mutations caused by RIP prevent successful translation of the genes coding for transposon movement proteins so they become inactive and can no longer replicate. This provides a very effective defense against transposon expansions in most fungi. In the powdery mildew fungi, the genetic machinery required for RIP was missing [43] and this likely allows unrestricted multiplication of transposons. For the rust fungi, no mechanisms for genome expansion were proposed or tested [44].

For *P*. *fijiensis*, *rid*, the only gene known to be required for RIP, is present and the reading frame appears to be intact. Repetitive sequences in the genome of *P*. *fijiensis* show high frequencies of the C to T transitions that are characteristic of RIP, so this phenomenon seems to be active. Because RIP is only active during meiosis, a possible explanation for fungi with extensive asexual phases could be that transposon expansion occurs during asexual reproduction and then is slowed by RIP during rare sexual reproduction. If transposons have expanded enough and RIP is not completely efficient, some intact copies of transposons could remain after meiosis to continue expanding during the next extended asexual phase. This explanation is possible in *P*. *fijiensis*; although it has been classically considered to be primarily reproducing by sexual mating, more recent studies suggest that asexual reproduction also plays an important role during epidemics [53,54]. Transposon expansion most likely occurs episodically when RIP or other mechanisms are relaxed, but when and how these episodes occur is currently unknown.

In *Z*. *tritici*, a different type of genome expansion occurred through the acquisition of a large set of dispensable chromosomes, referred to as the dispensome [17]. The *Z*. *tritici* dispensome contains at least eight chromosomes with no known function that appear to have been acquired by horizontal transfer from an unknown donor more than 10,000 years ago [17,55]. Such a large number of dispensable chromosomes with no known effects on fitness so far is unique among fungi. Many potential dispensable chromosomes were identified among the genomic scaffolds of *P*. *fijiensis* based on the characteristics of known dispensable chromosomes in *Z*. *tritici*. However, dispensability still has not been proven for *P*. *fijiensis*. None of the *P*. *fijiensis* linkage groups were missing in progeny isolates, one of the hallmarks of dispensable chromosomes in *Z*. *tritici* [56]. However, this is not surprising because none of the linkage groups corresponded to any of the putative dispensable scaffolds. If *P*. *fijiensis* does contain a dispensome, it is different from that in *Z*. *tritici* because there was almost no similarity between the dispensable chromosomes of *Z*. *tritici* and any of the scaffolds of *P*. *fijiensis*, or vice versa. This raises the intriguing possibility of separate events leading to horizontal transfer of large numbers of chromosomes between species in the Capnodiales and other fungi.

Electrophoretic karyotyping of *P*. *fijiensis* showed a remarkable level of variability among isolates, even those coming from the same population. This chromosome length and number polymorphism was also described previously in *P*. *fijiensis* [57] and in other fungi [58–60]. The mechanisms of such variation include chromosome rearrangements during meiotic recombination and the presence of dispensable chromosomes [61]. From a different perspective, it has been shown that chromosomal reshuffling can drive evolution of virulence in asexual plant-pathogenic fungi [62]; thus both sexual and asexual life cycles could be a source of chromosomal variation. This could constitute a mechanism of adaptation to environmental changes such as selective pressure from chemical fungicides. Field isolates karyotyped in this work were collected in Costa Rica in an area with a high level of fungicide applications.

Overall, *P*. *fijiensis* chromosomes are larger than those from other Ascomycetes, including its completely sequenced relative *Z*. *tritici* [17]. Remarkably, *P*. *fijiensis* contains a scaffold larger than 10 Mb, which is at the limit of PFGE resolution. Chromosomes of this size have been observed in other fungi [63–65] but they are not common. Medium-sized chromosomes were not found, similar to previous records for Mexican isolates [57]. Interestingly, the seven smallest main scaffolds, including the smallest calculated core chromosome (0.61 Mb) and six of the putative dispensable chromosomes did not appear in PFGE, as CIRAD86 did not show a chromosome smaller than 0.8 Mb. The total number of chromosomes separated by PFGE is at least 11, and probably up to 15 when possible co-migrating bands are counted separately, in addition to the five unresolved largest scaffolds.

The availability of a genome sequence enables the identification of genes that might be involved in pathogenicity, including those encoding putative effector proteins. Fungal effectors are proteins that aid pathogenicity, usually by subduing host defenses. However, these same proteins affecting pathogenicity also can be recognized by the host resistance proteins, triggering a defense response and making them advantageous or disadvantageous to the pathogen depending on the host genotype. Bioinformatic analysis of the *P*. *fijiensis* genome identified many putative effectors, including one that appears to be a homolog of the *Avr4* effector in the related Dothideomycete, *C*. *fulvum* [31]. The *P*. *fijiensis* putative *Avr4* homolog *PfAvr4* was on scaffold 4 adjacent to repeats of 617 and 2765 bp and a 6-kb AT-rich block with a GC content of 39.3%, similar to known effectors in other fungi, which often are in AT-rich regions [41,55]. Other genes in this region of the *P*. *fijiensis* genome were different from those in *Z*. *tritici* and *C*. *fulvum*, indicating little synteny among genes around the *PfAvr4* homologs in related species. Previous research showed that *PfAvr4* is a functional homolog of the *CfAvr4* virulence factor in *C*. *fulvum*, and that, despite a low amino acid identity of only 42%, it could be recognized by the Cf4 resistance protein to stimulate an HR in tomato [32]. However, whether *PfAvr4* could be recognized by banana cultivars had not been tested until now.

The response of the banana varieties to the PfAVR4 protein strongly suggests that it acts as an avirulence factor that is recognized by a resistant banana accession where it elicits an HR-like necrosis. Most probably this protein has a function similar to that of its homolog in *C*. *fulvum* where it is an effector that facilitates disease in susceptible cultivars of tomato and can be recognized by the Cf4 receptor in a resistant tomato cultivar to elicit the HR [31]. To our knowledge, this is the first fungal effector known to induce a cultivar-specific, HR-like necrosis in banana, suggesting that *M*. *acuminata* ssp. *burmannicoides* var. Calcutta 4 most likely has a functional R gene that recognizes PfAVR4, and which appears to be a functional homologue of *Cf4* in tomato. Additional experiments are needed to thoroughly test the hypothesis that var. Calcutta 4 contains an HR-inducing resistance gene effective against *P*. *fijiensis*. These could include analysis of progeny from controlled crosses between var. Calcutta 4 and a susceptible banana to test for co-segregation of necrosis induced by PfAVR4 and resistance to *P*. *fijiensis*, or deletion of *PfAvr4* to test whether the resulting mutant becomes virulent to var. Calcutta 4. However, these experiments would be challenging due to experimental limitations in this pathosystem: crosses in banana frequently suffer from segregation distortions due to the occurrence of translocations and functional analyses in *P*. *fijiensis* are not routine.

The banana var. Calcutta 4 has been a source of resistance against fungi, bacteria and nematodes in *Musa* breeding programs [66,67]. It is one of the most resistant accessions in field evaluations against *P*. *fijiensis* populations from around the world, with the exception of some isolates from the Pacific islands and Papua New Guinea [68], which are considered as the center of origin of the disease [5]. In addition, var. Calcutta 4 has shown resistance to crude extracts from *P*. *fijiensis* [69,70]. The identification of *PfAvr4* as a likely avirulence factor in var. Calcutta 4 provides a major advance for banana breeding programs aiming at increasing the level of resistance against black Sigatoka. Purified effector proteins can be used to identify other resistance genes and to facilitate rapid selection of resistant progeny from segregating populations. The current selection process in resistance breeding is inadequate as it exclusively relies on field evaluations, and is slow because black Sigatoka has a latent period of a month or longer and the banana cycling time, depending on the species, is approximately 10–15 months. Similar experiences slowed down resistance breeding in wheat to *Z*. *tritici* until the elucidation of its mating system showed single-gene inheritance of pathogenicity factors that facilitated more precise isolate characterizations and subsequent R-gene discovery [17,71–74]. Other potential genes involved in pathogenicity are discussed in supporting S2Text.

QoIs represent a class of fungicides that initially showed impressive efficacy against many plant pathogens [75]. However, resistance evolved rapidly and soon rendered the compound of little use in multiple pathosystems [76]. Diagnostic primers for the mitochondrial *cytb* gene showed that *P*. *fijiensis* is no exception, as the three commercial and frequently sprayed plantations were nearly or completely fixed for resistance. This is a remarkable shift compared to analyses performed during 2000–2003 when only part of the population was resistant [77]. Interestingly, the San Carlos population, which was not sprayed with fungicides, is still entirely sensitive. This result suggests limited genetic exchange between these populations that are separated by about 100 km. Nevertheless, even limited gene flow could have an impact on untreated areas. Because the selection pressure exerted by strobilurins is quite strong, the resistance frequency rapidly increases from a low number of resistant individuals to widespread resistance soon after fungicides are used, particularly since these compounds do not prevent sexual reproduction [78]. This prompted us to process the *P*. *fijiensis* genome sequence with a bioinformatics pipeline to develop VNTR markers for rapid PCR-based population analyses to compare with a priori neutral markers. Nonetheless, some genetic differentiation occurs between the populations as already described earlier in Costa Rica using microsatellite markers [79]. However, because populations have not yet reached mutation–drift equilibrium, gene flow could not be estimated using classical genetic models based on genetic differentiation [79]. Fortunately, new indirect [80] and direct [53] methods have been recently used to provide estimates of dispersal in *P*. *fijiensis* that could be integrated in theoretical and spatially explicit models to predict spatial patterns of fungicide resistance evolution under different management strategies.

The availability of the CIRAD86 genome sequence and the resequence data of CIRAD139 for *P*. *fijiensis* will blunt its continued threat to global production by facilitating the development of resistant cultivars in banana breeding programs. The rapid development of fungicide resistance and extreme variability of the *P*. *fijiensis* genome among isolates coupled with a high level of sexual reproduction make this pathogen highly adaptable to changing environmental conditions. Diversifying and increasing the level of host resistance in banana may be the only way to slow the devastation caused by this fungus in the future.

## Materials and Methods

### Fungal culture conditions and DNA extraction

*Pseudocercospora fijiensis* isolate CIRAD86 (*mat1-1* mating type, originating from Cameroon in 1988) was chosen for sequencing because it is the epitype for the species, has been the subject of intensive analyses previously and is one parent of an existing mapping population [81]. CIRAD139a (*mat1-2*, originating from Colombia in 1990) was used for resequencing. CIRAD86 is maintained at the CBS-KNAW Fungal Biodiversity Centre (CBS 120258).

Mycelia for DNA extraction were grown in 1L Erlenmeyer flasks containing 200 mL of PDB (potato dextrose broth; Becton Dickinson, NJ, USA) shaken at 120 rpm at 28°C. Mycelial mats produced during culture were filtered to remove the broth and lyophilized. Samples containing 50 mg of lyophilized mycelia were placed in 2 mL tubes and ground with a Hybaid Ribolyser (model n° FP120HY-230) for 10 s at 2500 rpm with a tungsten-carbide bead. DNA was extracted from the ground mycelia using the Wizard Magnetic DNA Purification system (Promega, Netherlands) for food according to instructions provided by the manufacturer.

### Genomic sequencing, genetic mapping, assembly and annotation

Whole-genome shotgun sequencing and assembly of the *P*. *fijiensis* genome were done using Sanger sequencing of three different-sized libraries (3- and 8-kb plasmids, and 40-kb fosmids) as described previously for *Z*. *tritici* [17] and other species [82]. The initial version 1 assembly was improved by aligning the physical scaffolds to a genetic linkage map constructed using Joinmap V 4.0 software [83] to analyze the segregation data for 322 markers that were scored on 135 progeny of the cross between isolates CIRAD86 and CIRAD 139A [81]. For each molecular locus, a goodness-of-fit analysis was performed to test for deviation from the expected 1:1 segregation ratio at a 1% significance level. Linkage groups were established using a minimum LOD score of 9.0 and final mapping was achieved by combining two or more linkage groups belonging to the same chromosome. The order of the markers on each chromosome was determined using a minimum LOD score of 1.0, recombination threshold of 0.4, jump of 5.0, ripple value of 1 (default) and Haldane’s mapping function as parameters. In cases of uncertainty, some markers were removed and the order was recalculated until a more stable order was achieved.

Three methods were used to identify the *P*. *fijiensis* repetitive sequences. Repeated sequences in the genome were identified *de novo* using RECON [84] and the k-mer based method RepeatScout [85]. A custom set of repeats and the RepBase Update library of 234 fungal repeats [86] were then used to mask the *P*. *fijiensis* genome using RepeatMasker (http://www.repeatmasker.org/) [87].

Repeat families with 10 or more elements identified by RepeatScout were annotated and classified into categories based on the presence of protein domains (BLAST [88]). Structural features including Long Terminal Repeats (LTRs) and Terminal Inverted Repeats (TIRs) were verified using the EMBOSS [89] software package. Sequences with no known proteins or structural features were grouped into the unclassified category.

Identification and annotation of protein-coding genes were performed using the JGI Annotation Pipeline, which takes multiple inputs (scaffolds, ESTs, and known genes), runs several analytical tools for gene prediction and annotation, and deposits the results in the JGI fungal genome portal MycoCosm (http://jgi.doe.gov/fungi) [90] for further analysis and manual curation.

Several gene-prediction programs falling into three general categories were used to annotate the repeat-masked assembly as described by Ohm et al. [19]. The resulting set of putative genes was then filtered for the best models based on EST and similarity support to produce a non-redundant representative set. This representative set of filtered gene models from the automated annotation pipeline was subject to further analysis and manual curation as described by Goodwin et al. for *Z*. *tritici* [17] and by Ohm et al. [82] for more recently sequenced species. Measures of model quality included proportions of the models complete with start and stop codons (88% of models), those that were consistent with ESTs (30% of models) and those supported by similarity with proteins from the NCBI NR database (74% of models) as summarized in S8 Table.

Functional annotations for all predicted gene models were made using SignalP [91], TMHMM [92], InterProScan [93], and BLASTp [88] against the nr, SwissProt (http://www.expasy.org/sprot/), KEGG [94] and KOG [95] databases as described by Ohm et al. [19]. Multigene families were predicted with the Markov clustering algorithm (MCL) [96] to cluster the proteins using BLASTp alignment scores between proteins as a similarity metric. Functional annotations are summarized in S9 Table. Manual curation of the automated annotations was performed using the web-based interactive editing tools of the JGI Genome Portal to assess predicted gene structures, assign gene functions, and report supporting evidence. Gene models predicted by the JGI annotation pipeline were also analyzed using the program Blast2GO [97] with an E-value of < 10^−6^. Blast2GO assigns GO terms based on the BLAST definitions. Comparisons between groups of genes for enrichment of GO terms were done by using Fisher’s exact test implemented in the Blast2GO program.

Potential secreted proteins were identified with a python script made to run all gene models through SignalP 3.0 [98] and subsequently filtered for proteins that had no transmembrane domains, no signal anchor motifs, were fewer than 300 amino acids in length and had at least four cysteine residues. The gene models that fulfilled these criteria were considered as potential SSPs.

For re-sequencing of isolate CIRAD 139A, a paired-end library was made using the standard Illumina library prep protocol with NEB reagents. Average insert size of the library was 272 base pairs. Sequencing was done on an Illumina GAIIx in one lane of a 54-cycle paired-end run using 36-cycle version 5 SBS Kits. The flow cell was built using a version 4 paired-end cluster generation kit. Eventually, 37 million reads were obtained for a total yield of 4 gigabases. Paired reads were aligned to the *P*. *fijiensis* v2 Assembly reference scaffolds using GSNAP (2010-03-09 release), allowing up to 3 mismatches or 1 indel and with end trimming enabled. Uniquely aligned reads were then used to call variant sites using the Alpheus pipeline, requiring that a variant have support from at least two reads with an average quality of bases of at least phred 10 and at least 80% of the reads covering the site calling that variant. Nonsynonymous SNP differences were assessed against the coding regions in the *P*. *fijiensis* v2 Frozen Gene Catalog 20100402.

To survey the non-synonymous SNPs in the annotated protein set of CIRAD86, a simple analysis of functional bias in variant proteins was conducted using a ranking comparison approach. All genes were ranked based on their non-synonymous SNP count (normalized for coding sequence length) and two selected gene sets were compared with the whole-genome set. The two sets used for comparison were the 1500 most-variant proteins (Set V) and the 1500 least-variant proteins (Set C), taken from the list of genes ranked by non-synonymous SNP count. The annotation files for the *P*. *fijiensis* v2 Frozen Gene Catalog 20100402 were used as the source of GO terms for the genes. The ranked frequency of occurrence of GO terms in the gene annotations for the whole genome was compared with those for Sets C and V.

### Repetitive element analysis

For each repetitive element family, a subset of elements with lengths within 50% of the longest element was aligned using clustalX [99]. These alignments were submitted to RIPCAL [28] to determine the dinucleotide bias observed in repetitive elements. RIPCAL estimates 'RIP dominance' for each dinucleotide containing a cytosine. It is the ratio of a given dinucleotide (e.g., CA) to the sum of the other three dinucleotides (CG/CC/CT).

To test for isochores in the *P*. *fijiensis* genome, a contiguous stretch of sequence with an arbitrarily chosen average GC content of less than 45% was categorized as an AT block. Custom python scripts were used to calculate the percent GC across the genome, to generate AT blocks and to calculate the average percent GC across the AT blocks and those fewer than 500 bp apart were merged into blocks of at least 1 kb in length, which were retained and analyzed for their composition and distribution of repetitive sequences and genes.

To estimate and compare the amount of RIP between the AT-rich blocks and the rest of the genome, a custom python script was written using a 500-bp sliding window with a step size of 100 bp. The amount of RIP was calculated as an index (CpA+TpG)/(ApC+GpT) and estimated separately for each of the AT-rich versus AT-poor regions in the genome. The RIP index measures the depletion of the RIP targets CpA and TpG; thus, lower values of (CpA+TpG)/(ApC+GpT) are indicative of a higher degree of RIP [28].

To estimate the ages of transposon insertions, LTR retrotransposons were identified and annotated using the LTRharvest [100] and LTRdigest [101] modules in GenomeTools [102]. LTR sequences from these elements were aligned using ClustalW [99] and manually curated to estimate the numbers of mutations that had accumulated over time. All transition mutations were ignored in this analysis to remove the bias caused by RIP. Age of the LTR retrotransposons was calculated using the average rate of 1.09 × 10^−9^ substitutions/site/year as proposed for fungal sequences [103].

To test whether the transposons in the *P*. *fijiensis* genome were unique, a comparison was made to transposable elements (TEs) in the genome of *C*. *fulvum*, the only other sequenced fungus in the Capnodiales with an expanded genome. RepeatMasker [85] was used to mask the *C*. *fulvum* genome using the repeat database from the *P*. *fijiensis* genome. The resulting file was parsed using the 80:80 rule of Wicker et al. [27], i.e., 80% identity across 80% length to identify repeats in common between the two genomes. Another run was done at a 70:70 cutoff to allow for greater divergence generated by RIP.

### Generation and analysis of EST sequences

The CIRAD86 strain was grown in three culture media for production of cDNA libraries: yeast-glucose broth as a rich medium (10 g of yeast extract and 30 g of glucose per liter); minimal nutrient medium (1 g of KH_2_PO_4_, 1 g of KNO_3_, 0.5 g of MgSO_4_·7H_2_O, 0.5 g of KCl, 0.2 g of glucose, 0.2 g of sucrose per liter); and minimal nutrient medium without a nitrogen source (as above but without KNO_3_). Fungal mycelia were grown in each medium at 25°C for 10 days with a photoperiod of 12 hours using cool-white fluorescent light on a rotary shaker at 100 rpm. Mycelia derived from all three *in vitro* conditions were harvested by filtration and ground in liquid nitrogen. The RNA was isolated by the trizol method with the RNeasy kit (Qiagen, Netherlands) with 2 g of starting material. RNA quality and quantity were assessed by spectrophotometer and by gel electrophoresis according to standard procedures. For cDNA library construction, first-strand cDNA synthesis was done using polyA+ RNA, reverse transcriptase (SuperScriptII (Invitrogen, CA, USA)) and an oligo dT-*Not*I primer (5' GACTAGTTCTAGATCGCGAGCGGCCGCCCT15VN 3'). Second-strand synthesis was done by *E*. *coli* DNA ligase, polymerase I and RNaseH before end repair with T4 DNA polymerase. The *Sal*I adaptor (5' TCGACCCACGCGTCCG and 5' CGGACGCGTGGG) was ligated to the cDNA and digested with *Not*I before selecting the size range by gel electrophoresis. Sizes were 0.6–2 kb and 2–10 kb. The cDNA of *P*. *fijiensis* grown in yeast-glucose medium was divided into libraries CBBT and CBHU (0.6-2kb) and CBHT (2-10kb). The cDNA from culture on minimal nutrient medium was divided into libraries CBBW and CBHX (0.6-2kb) and CBBU and CBHW (2-10kb), and for the libraries of culture on minimal nutrient medium without nitrogen source, cDNA was divided into libraries CBBX and CBHY(0.6-2kb). The size-selected inserts were cloned into the pCMVSPORT6 vector (Invitrogen) and digested with *Sal*I and *Not*I. Ligated vectors were transformed into ElectroMAX T1 DH10B cells (Invitrogen).

Sequence reads from cDNA libraries were trimmed of vector, linker, adapter, poly-A/T, and other artifact sequences with the Cross-match software. Internally developed software at the JGI-DOE identified short patterns and low-quality regions (Q15). The longest high-quality region of each read was counted as an EST. Clustering of ESTs was performed based on pairwise alignments generated using the Malign software, a modified version of the Smith–Waterman algorithm [104], which was developed at the JGI for use in whole-genome shotgun assembly. ESTs sharing an alignment of at least 98% identity with 150-bp overlap were assigned to the same cluster. For each cluster of EST sequences, a consensus sequence was generated by running the Phrap software [105,106].

### Functional analysis of a putative effector protein

Plantlets of *M*. *acuminata* ssp. *burmannicoides* var. Calcutta 4 (recognized as a resistant standard for BLSD) were multiplied and rooted *in vitro*, whereas Cavendish “Grand Naine” tissue culture plants were hardened for three to four weeks in a greenhouse environment. Subsequently, all plantlets were grown for three months (small plants), and some plants of var. Calcutta 4 and “Grand Naine” were grown for eight months (large plants) in a controlled-environment greenhouse compartment at 25°C with a relative humidity of >80% and 16 hours of light per day.

Plants of tomato (*Lycopersicum esculentum*) cv. Moneymaker (MM), which has no known *Cf* resistance genes (*Cf0*), or an isogenic line previously transformed with the *Cf4* resistance gene were grown under greenhouse conditions as described by Stergiopoulos et al. [32] during 3–4 weeks.

The mature protein from the *P*. *fijiensis* putative effector gene *PfAvr4* was produced heterologously by culturing *Pichia pastoris* isolate GS115 in a fermentor as described previously [107]. Following production in the fermentor, the protein was further purified from excess liquid medium and smaller proteins by filtration through a 3-kDa membrane (Amicon Ultra-15 Centrifugal filter unit, Millipore, USA).

Infiltration of the complete fermentor product or purified PfAVR4 protein into banana and tomato leaves was done by injection with a 1-mL syringe with no needle. Infiltrations on banana leaves were done at the original concentration and a six-fold higher concentration. For tomato, all infiltration materials were diluted fifty or seventy-five times prior to infiltration. Samples of the fermentor medium and water were infiltrated separately as negative controls for all plants. At each infiltration point, the observed water soaking of tissue was marked with a permanent marker. Observations were recorded with an Olympus C-8080 digital camera at four and 10 days post infiltration (dpi) on banana leaves, and at 6 dpi for tomato plants. Protein preparation and controls were infiltrated in at least two banana leaves from each genotype in small and large sizes, with at least 3 repetitions per leaf. Infiltrations in tomato plants were performed in at least 4 leaves with one repetition.

### Thermal denaturation assays

The thermal denaturation method of Marmur and Doty [108], performed basically as described by Smith et al. [109], was used to estimate G+C contents of DNA from *P*. *fijiensis* isolate CIRAD86 (CBS120258) plus that from the closely related banana pathogens *P*. *musae* (isolate UQ430; CBS121371) and *P*. *eumusae* (isolate CBS122457) as well as the previously sequenced *Z*. *tritici* isolate IPO323 (CBS115943) [17]. Genomic DNA was isolated from cultures grown in PD broth at 25°C on a rotary shaker (150 rpm) following the procedure described by Raeder and Broda [110] and was dissolved in 0.1X SSC. Melting curves were obtained on a Perkin-Elmer λ25 spectrophotometer equipped with a thermal programmer. The G+C contents were calculated from the T_m_ values (melting/transition temperature) derived from the peaks of the first derivatives of the melting curves [111]. DNA from *Candida parapsilosis* isolate CBS604 (T_m_ in 0.1 x SSC, 70.6°C) [3] was used as a calibration control. Determinations were performed at least twice for each isolate.

### Phylogenetic analysis

Phylogenetic analysis showing the placement of species within the Capnodiales with expanded genomes was done using Internal Transcribed Spacer regions (ITS). DNA sequences were downloaded from GenBank with the following accession numbers: AF181692 for *Z*. *tritici*, EU514233 for *P*. *eumusae*, EU514265 for *P*. *musae* and EU514248 for *P*. *fijiensis* or obtained from genome data available at the Fungal Genome portal at JGI. Sequence alignment was done using MUSCLE [112] and the phylogenetic tree was generated with MEGA 6.0 [113] using a Maximum Likelihood statistical method and the Tamura 3-parameter substitution model. Support for the nodes of the tree was estimated by bootstrapping with 1000 replications.

### Electrophoretic karyotyping

Isolates of *P*. *fijiensis* grown for 3 weeks in PD broth at 28°C, 150 rpm, were blended and grown for 48 hrs in the same medium at 20% strength amended with 1 μM tricyclazole. Decanted culture was washed with 1 M sorbitol, and added to 40 mL of OM buffer (1.2 M MgSO4, 10 mM K phosphate, pH 5.8 with 700 mg of glucanase (Sigma, Germany), 256 mg of yatalase (Takara, Japan), 7500 U of β-glucuronidase (Sigma) and 0.8 g of driselase (Sigma)) in a ratio of ~1:3 (mycelium:buffer). The enzymatic treatment was incubated at 33°C and shaken at 50 rpm for 4.5 hrs.

Protoplasts were filtered through a plastic mesh of 30 μm and washed 3 times with 1 M sorbitol in sterile conditions. When the concentration was at least 1 × 10^8^ per mL, protoplasts were embedded in low-melting point (SeaKem Gold) agarose at a final concentration of 0.5%. Agarose plugs were treated with proteinase K as described previously [114], washed with cold 50 mM EDTA, and kept in the same solution at 4°C until used.

Chromosomes of *P*. *fijiensis* were separated in a CHEF DR-II system (Bio-Rad, Netherlands). Small chromosomal bands were discriminated as described before [57] using the chromosomes of *Saccharomyces cerevisiae* (Bio-Rad) as a high molecular weight (HMW) standard. Large chromosomes were separated in a 0.8% low-melting point (SeaKem Gold) agarose gel, with 0.5% TBE buffer at 11°C, and 50 V for 195 hrs with switching times from 4800 to 1800 sec, and 24 hrs from 1800 to 1300 sec, followed by 20 hrs at 60 V from 1300 to 800 sec, and finally 27 hrs of 800 to 600 sec at 80 V. HMW standards were *Schizosaccharomyces pombe* and *Hansenula wingei* chromosomes (BioRad). Agarose gels were stained with SYBRGold (Invitrogen) and destained in water for 30 and 20 min, respectively, observed under a UV transilluminator and recorded with an Eagle Eye II (Stratagene) still video system.

### Whole-genome comparisons and synteny analyses

Two tools, Circos [115] and MUMmer [116], were used for structural analysis of the *P*. *fijiensis* genome. A nucleotide-based similarity search was done between the masked *P*. *fijiensis* and *Z*. *tritici* genomes and visualized using Circos [115], whereas protein comparisons between the masked genomes were done using Promer [116]. Proteins with greater than 60% identity were reported.

Proteins in *P*. *fijiensis* and *Z*. *tritici* with at least 50% amino-acid identity and match length were grouped as orthologs using OrthoMCL [117] and synteny blocks were determined using Orthocluster [118]. The *Z*. *tritici* protein dataset also was compared to two other phylogenetically distant Dothideomycetes in the order Pleosporales, *P*. *nodorum* and *P*. *tritici*-*repentis*.

### Fungicide sensitivity and population analyses

To analyze the frequencies of molecular marker alleles and fungicide resistance within populations, four farms in Costa Rica were sampled during 2008 (S8 Fig). San Pablo, Zent, and Cartagena are located in Limón province, where bananas are grown at high density on large plantations and diseases are controlled by using chemical fungicides. These farms are located in the main Costa Rica banana production area with approximate sizes of 285, 342 and 64 ha, respectively. A fourth farm, San Carlos (0.5 ha), is located in Alajuela province and is isolated geographically from the principal banana-production area. Leaf tissue was collected from ten banana plants from each farm. Ascospores were discharged from the pseudothecia onto water agar [119] and single ascospores were transferred immediately to 15 x 100-mm petri dishes filled with potato dextrose agar (PDA). Between eight to ten ascospores from each sample point were placed on each dish of PDA. After 4 days colonies were transferred to Mycophil agar (Becton Dickinson Microbiology Systems, Cockeysville, MD) and incubated for 15 days at 25°C under continuous fluorescent light for colony growth and conidial production. Eventually, 649 isolates were collected and analyzed for phenotypic and molecular variability.

To obtain DNA for population genetics analyses, mycelia of 190 isolates from each of the three commercial plantations and of 95 isolates from the San Carlos population were harvested and lyophilized for 24 hours. Genomic DNA was extracted using the Wizard Magnetic DNA Purification System for Food Kit (Promega, Madison, WI, USA) according to the manufacturer’s instructions and 2 μL per sample were quantified using a NanoDrop ND-1000 Spectrophotometer (NanoDrop Technologies, Wilmington, DE, USA). Mating type (*mat*) PCR assays [120] were performed in a 50-μL total volume containing 50 ng of template genomic DNA, 2 mM MgCl_2_, 600 μM dNTPs, 5 μM of each primer, and 0.4 U of Taq DNA polymerase (Roche, Mannhein, Germany). Temperature cycling was carried out with the following program: 94°C for 2 min, 40 cycles of 94°C for 1 min, 70°C for 30 s and 72°C for 1 min, and a final elongation period of 10 min at 72°C.

Analysis of VNTR markers was done as reported previously [121]. For genotyping strobilurin resistance, primers were developed on the basis of the G143A mutation in the *cytb* gene [122] to identify sensitive and resistant *P*. *fijiensis* field strains (S10 Table) in 20-μL aliquots containing 50 ng of template genomic DNA, 2 mM MgCl_2_, 600 μM dNTPs, 5 μM of each primer and 0.4 U of Taq DNA polymerase (Roche, Mannhein, Germany). Temperature cycling was conducted with the following program: 94°C for two min, 40 cycles of 94°C for one min, 70°C for 30 s and 72°C for one min, and a final elongation period of 10 min at 72°C. Amplicons were separated by electrophoresis using 1% (for *mat* and *cytb* assays) or 3% (for VNTRs) agarose gels containing 0.3 μg/mL ethidium bromide, in 0.5× TBE buffer at 120 V for approximately 1 h (for *mat* and *cytb* assays) or 5 h (for VNTRs) and were visualized and photographed using a UV transilluminator and Eagle Eye II (Stratagene) still video system.

To analyze the data, frequencies of the two mating types within each population and in the overall sample were tested for deviation from a 1:1 ratio with χ^2^ tests. A molecular multilocus haplotype was constructed for each isolate by combining the allelic data at all five VNTR loci. Gene diversity within each population (*H*_*S*_) in total and by locus was calculated using GenAlEx 6.4 [123]. Total diversity over the entire sample (*H*_*T*_), mean gene diversity within populations (*H*_*S*_), genetic differentiation among populations (*G*_*ST*_) and the corrected, standardized measure of genetic differentiation (*G”*_*ST*_) were calculated using GENODIVE Beta version 2.0 [124]. In all cases, *H*_*T*_ and *H*_*S*_ refer to the unbiased estimates as developed by Nei [125]. Pairwise estimates of *G”*_*ST*_ and of Jost’s differentiation (*D*) [126] also were calculated with GENODIVE. Multilocus haplotype diversity was calculated with multilocus (http://www.bio.ic.ac.uk/evolve/software/).

## Supporting Information

S1 Text Additional information on genome sequencing, assembly and EST support. (DOCX)

S2 Text Additional potential pathogenicity-related genes present in the *P*. *fijiensis* genome. (DOCX)

S1 Fig The strong positive association between RIP index (where a high index value indicates low RIP) and GC content shows that RIP in *P*. *fijiensis* is mostly restricted to repetitive elements rather than genes. (TIF)

S2 Fig The RIP index in genes and repeats in AT-rich and–poor regions of the *P*. *fijiensis* genome. RIP is mostly absent from the genes but highly prevalent among the repeated elements of the genome.
(TIF)

S3 Fig Separation of small size chromosomal bands by electrophoretic karyotyping of five field isolates and the CIRAD139A strain of *Pseudocercospora fijiensis*. Lane 1, chromosomes from *Saccharomyces cerevisiae* as high-molecular-weight (HMW) marker; lanes 2 to 6, different field isolates from the Cartagena farm; lane 7, the CIRAD139A strain. Marker sizes are in Kb.
(TIF)

S4 Fig Dot plot showing mesosynteny between the scaffolds of *Pseudocercospora fijiensis* and *Septoria musiva*. (TIF)

S5 Fig Dot plot showing mesosynteny between the scaffolds of *Pseudocercospora fijiensis* and *Zymoseptoria tritici*. (TIF)

S6 Fig Dot plot showing mesosynteny between the scaffolds of *Septoria musiva* and *Dothistroma septosporum*. (TIF)

S7 Fig Small secreted proteins in the genome of *Pseudocercospora fijiensis* compared to those in the genomes of three other Dothideomycetes. (TIF)

S8 Fig Locations of farms in Costa Rica that were sampled to obtain isolates of *Pseudocercospora fijiensis* for analyses of mating type, fungicide resistance and population genetics. Farms Cartagena, San Pablo and Zent are in a major banana-production area and are sprayed heavily with fungicides; the San Carlos farm is in an area of plantain production (mostly resistant to *P*. *fijiensis*) and is not sprayed with fungicides.
(DOCX)

S1 Table Scaffold sizes, and identification of potential dispensable chromosomes in the genome of *Pseudocercospora fijiensis* by comparison with the characteristics of those known from *Zymoseptoria tritici*. (DOCX)

S2 Table Comparison of annotated genes in the version 1 and 2 assemblies of the *Pseudocercospora fijiensis* genome. (DOCX)

S3 Table Summary of G+C contents derived from the melting-curve analyses of DNA extracted from isolates of *Pseudocercospora fijiensis*, *P*. *eumusae*, *P*. *musae* and *Zymoseptoria tritici*. (DOCX)

S4 Table Next-generation resequencing information for *Pseudocercospora fijiensis* isolate CIRAD139A and numbers of Single-Nucleotide Polymorphisms (SNPs) compared with the reference genome of isolate CIRAD86 V2.0. (DOCX)

S5 Table Analysis of polymorphism type [Single-Nucleotide Polymorphisms (SNPs) and insertion/deletion (indels)] and frequency among the 21 largest scaffolds of the sequenced *Pseudocercospora fijiensis* isolate CIRAD86 and the re-sequenced isolate CIRAD139A. (DOCX)

S6 Table Genotyping strobilurin resistance and mating type in four Costa Rican populations of *Pseudocercospora fijiensis*. (DOCX)

S7 Table Estimates of genetic differentiation^a^ (Jost’s D above the diagonal and the corrected, standardized genetic differentiation G″_ST_ below the diagonal) between pairs of populations of *Pseudocercospora fijiensis* sampled from four banana plantations in Costa Rica^b^. (DOCX)

S8 Table Percent of gene models supported by different kinds of evidence in the initial automated annotation of the genome of *Paracercospora fijiensis*. (DOCX)

S9 Table Automated assignment of the 13,107 genes in the genome of *Pseudocercospora fijiensis* to broad functional categories. (DOCX)

S10 Table Primers used for population genetic diversity and fungicide sensitivity assays in four *Pseudocercospora fijiensis* populations in Costa Rica. (DOCX)
