# Correction: Combating a Global Threat to a Clonal Crop: Banana Black Sigatoka Pathogen *Pseudocercospora fijiensis* (Synonym *Mycosphaerella fijiensis*) Genomes Reveal Clues for Disease Control

**DOI:** 10.1371/journal.pgen.1006365

**Published:** 2016-10-04

**Authors:** The PLOS Genetics Staff

## Notice of Republication

This article was republished on September 14^th^, 2016, to correct an error in the XML that prevented the Corresponding Author information for Gert H. J. Kema from displaying correctly, and to remove tracked changes from the following Supporting Information files: S1, S2, S3, S4, S5, S7, and S10 Table; S1 and S2 Text; and S8 Fig. The publisher apologizes for the errors. The PDF version of the article is unchanged.

## References

[1] Arango Isaza RE, Diaz-Trujillo C, Dhillon B, Aerts A, Carlier J, Crane CF, et al (2016) Combating a Global Threat to a Clonal Crop: Banana Black Sigatoka Pathogen *Pseudocercospora fijiensis* (Synonym *Mycosphaerella fijiensis*) Genomes Reveal Clues for Disease Control. PLoS Genet 12(8): e1005876 doi: 10.1371/journal.pgen.1005876 27512984 10.1371/journal.pgen.1005876 PMC4981457

