# Supplementary material for: Combating a Global Threat to a Clonal Crop: Banana Black Sigatoka Pathogen Pseudocercospora fijiensis (Synonym Mycosphaerella fijiensis) Genomes Reveal Clues for Disease Control
Source: PLoS Genet. 2016 Aug 11;12(8):e1005876. doi: 10.1371/journal.pgen.1005876 (PMC4981457; doi:10.1371/journal.pgen.1005876)
Supplement: S1 Text — (DOCX) [file pgen.1005876.s001.docx]

# Text S1. Additional information on genome sequencing, assembly and EST support.

**Genome sequencing and assembly**

*Pseudocercospora fijiensis* isolate CIRAD86 was sequenced using Sanger sequencing on ABI 3730XL capillary machines. Three libraries with different sized inserts (3- and 8-kb plasmids, and 40-kb fosmids) were used as templates for the plasmid subclone sequencing process and both ends were sequenced. After trimming sequences for vector sequences and eliminating those with low quality, a total of 867,068 reads was assembled into 382 main genome scaffolds using a modified version of Arachne [1]. The resulting *P. fijiensis* v1.0 draft assembly totaled 73.6 Mb at an average read depth coverage of 7.11×. Almost 84% of the genome was contained in 10 scaffolds that were each at least 4.0 Mb in length (Table S1).

Inclusion of the genetic map data to facilitate assembly of the physical genome sequence involved sequencing 288 markers and aligning them to the version 1 draft assembly. Among the 288 markers sequenced, 262 were mapped to the version 1 genome assembly while 26 markers did not map. The improved version of the genome sequence has an estimated size of 74 Mb, assembled into 56 scaffolds covering more than 99% of the genome. The largest scaffold is 11.8 Mb in length and 28 scaffolds (99.8.%) are larger than 50 Kb. Only 0.6% of sequence bases are estimated to be in gaps as compared to 8.9% in version 1.0.

Annotation of the v1.0 assembly using a variety of similarity-based and *ab initio* gene predictors [2] yielded 10,316 genes. Annotation of the v2.0 assembly was with the same annotation pipeline but with additional filtering by mapping of the v1.0 gene catalog along with its manual curations. After filtering for EST support completeness and similarity to other species, 13,107 genes were structurally and functionally annotated. The average gene length in the version 2 assembly is 1,833 nt with 3.62 exons per gene; 88%, are complete with start and stop codon, 74% have similarity support, and 49% have Pfam domains (Table S2). Most of the gene models (96%) are located in 12 scaffolds, numbers 1-10, 12 and 19. Gene density in these 12 scaffolds varies from 151 to 229 per Mb and drops from 2.2 to 94 genes per Mb for the remaining scaffolds larger than 0.5 Mb (Table S1).

**EST support**

Sequencing of nine cDNA libraries from three *in vitro* conditions generated 32,394 ESTs: 10,733 from growth in rich medium (library MFEST-3); 11,685 from growth in minimal medium with nitrogen (library MFEST-4); and 9,976 from growth in minimal medium without nitrogen (library MFEST-5). Average read length was 711.8 bp and percent GC was just over 53%, corresponding to the high-GC peak seen in thermal-denaturation assays.

Clustering of ESTs from all libraries using Cap3 yielded 3,306 contigs and 3,164 singletons. Mapping of the ESTs to the version 2 genome assembly provided support for 5,663 of the predicted gene models; 99% of these are located in 13 scaffolds. Some scaffolds such as 11 and 14 had a very low density of genes and mapped ESTs (Table S1).

Differences in the relative abundance of EST sequences among the three libraries provide some clues about gene expression under each culture condition. Under rich conditions (library MFEST-3), genes that were more abundant included those with GO terms related to metabolic processes such as carbohydrate catabolic processes (GO:0046365, GO:0019320), lipid metabolic processes (GO:0006629 GO:0045834), dicarboxylic acid metabolic process (GO:0043648) and small-molecule biosynthetic process (GO:0044281), among others. Some interesting genes expressed exclusively under rich conditions in library 3 include a polyketide synthase, a D-3-phosphoglycerate dehydrogenase related to cytochrome p450 of *Neurospora crassa*, a Ctr copper transporter family protein (*Aspergillus clavatus* NRRL 1), and an ABC multidrug transporter mdr2 protein.

Genes with more abundance in minimal medium in the presence of nitrogen (library MFEST-4) had GO terms related to membrane fraction type proteins (GO:0005624), alcohol metabolic process (GO:0006066), binding (GO:0005488) and biosynthetic processes (GO:0009058, GO:0046364, GO:0019319). Interesting genes in MFEST-4 include a homolog of hydrophobin 1, which is the highest-expressed gene in this library, an alcohol oxidase and an extracellular cell wall glucanase. Several highly expressed genes in library MFEST-4 also were expressed in library MFEST-5 including the hydrophobin 1 and the alcohol oxidase. Enriched GO terms in library MFEST-5 included, among others, drug transmembrane transporter activity (GO:0015238 GO:0015893), ion transmembrane transporter activity (GO:0015075, GO:0006812, GO:0006811 and others) and several terms related to nucleotide binding activities (GO:0001882, GO:0032553).

**References**

1. Jaffe DB, Butler J, Gnerre S, Mauceli E, Lindblad-Toh K, Mesirov JP, et al. Whole-Genome Sequence Assembly for Mammalian Genomes: Arachne 2. Genome Res. 2003;13: 91–96. doi:10.1101/gr.828403

2. Ohm RA, Feau N, Henrissat B, Schoch CL, Horwitz BA, Barry KW, et al. Diverse Lifestyles and Strategies of Plant Pathogenesis Encoded in the Genomes of Eighteen Dothideomycetes Fungi. PLoS Pathog. 2012;8: e1003037. doi:10.1371/journal.ppat.1003037
