# Supplementary material for: Combating a Global Threat to a Clonal Crop: Banana Black Sigatoka Pathogen Pseudocercospora fijiensis (Synonym Mycosphaerella fijiensis) Genomes Reveal Clues for Disease Control
Source: PLoS Genet. 2016 Aug 11;12(8):e1005876. doi: 10.1371/journal.pgen.1005876 (PMC4981457; doi:10.1371/journal.pgen.1005876)
Supplement: S2 Text — (DOCX) [file pgen.1005876.s002.docx]

### **Text S2. Additional potential pathogenicity-related genes present in the *P. fijiensis* genome**.

Other potential genes involved in pathogenicity could be identified from EST support under different conditions. Several interesting genes were expressed in the libraries collected in minimal medium with and without nitrogen, which resemble the conditions that the fungus might face in a natural environment, including a hydrophobin 1 and an alcohol oxidase. An alcohol oxidase is a pathogenicity factor in *Cladosporium fulvum*[1], and the class I hydrophobin, MPG1, is involved in the formation and attachment of appressoria in the rice pathogen *Magnaporthe oryzae* [2]. Hydrophobins allow fungi to escape their aqueous environment and mediate attachment of hyphae to hydrophobic surfaces, which is important during the initial steps of fungal pathogenesis, where the fungus must attach to the hydrophobic surface of the host before penetration and infection [3]. Because *P. fijiensis* does not penetrate directly it must grow on the surface of a leaf until it finds a stoma. The hydrophobin protein possibly could be involved in attachment to the host during its epiphytic phase until it can locate a suitable stoma for penetration.

1. Additional potential pathogenicity-related genes were identified through analysis of the pathways for melanin biosynthesis. Melanin is essential for successful host penetration by several plant-pathogenic fungi [4–6]. Although there are different types of fungal melanin, many pathogenic fungi, e.g., *Magnaporthe oryzae*, *Verticillium dahliae*, *Blastomyces dermatitidis* and *Sporothrix schenckii*, synthesize their pigments from acetate through the 1,8-dihydroxynaphthalene (DHN)-melanin pathway [7–9]. Both *Z. tritici* and *P. fijiensis* may use this pathway for melanin biosynthesis because the necessary genes are present in their genomes. However, in *Z. tritici* melanin does not appear to be important for pathogenicity since melanin-deficient mutants were still pathogenic [10], while mutants with increased melanin production showed reduced pathogenicity [11]. The importance of melanin for pathogenicity of *P. fijiensis* is not known.
2. The DHN-melanin pathway may be important for *P. fijiensis* even if melanin is not involved in its pathogenicity. In addition to melanin, this pathway is used for the synthesis of juglone and 2,4,8-tetrahydroxytetralone, both of which are produced by *P. fijiensis* in large amounts [12,13] and are phytotoxic to the banana plant, most probably by acting on chloroplasts [14]. Knowing the sequences of all of the genes involved in the synthesis of these metabolites will open the way for functional analyses, allowing tests of whether they have a role in pathogenesis. The only knowledge in this respect is the virulence test of two pigment-deficient isolates [15]. Both isolates were capable of penetrating, but only one of them was virulent.

**References**

1. Segers G, Bradshaw N, Archer D, Blissett K, Oliver RP. Alcohol Oxidase Is a Novel Pathogenicity Factor for *Cladosporium fulvum* , but Aldehyde Dehydrogenase Is Dispensable. Mol Plant Microbe Interact. 2001;14: 367–377. doi:10.1094/MPMI.2001.14.3.367

2. Talbot NJ, Ebbole DJ, Hamer JE. Identification and characterization of MPG1, a gene involved in pathogenicity from the rice blast fungus *Magnaporthe grisea*. Plant Cell Online. 1993;5: 1575.

3. Hektor HJ, Scholtmeijer K. Hydrophobins: Proteins with potential. Curr Opin Biotechnol. 2005;16: 434–439.

4. Kubo Y, Suzuki K, Furusawa I, Yamamoto M. Melanin biosynthesis as a prerequisite for penetration by appressoria of *Colletotrichum lagenarium*: site of inhibition by melanin-inhibiting fungicides and their action on appressoria. Pestic Biochem Physiol. 1985;23: 47–55.

5. Rasmussen J, Hanau R. Exogenous scytalone restores appressorial melanization and pathogenicity in albino mutants of *Colletotrichum graminicola*. Can J Plant Pathol. 1989;11: 349–352.

6. Wolkow P, Sisler H, Vigil E. Effect of inhibitors of melanin biosynthesis on structure and function of appressoria of *Colletotrichum lindemuthianum*. Physiol Plant Pathol. 1983;23: 55–71.

7. Bell AA, Wheeler MH. Biosynthesis and functions of fungal melanins. Annu Rev Phytopathol. 1986;24: 411–451.

8. Butler M, Day A. Fungal melanins: a review. Can J Microbiol. 1998;44: 1115–1136.

9. Romero-Martinez R, Wheeler M, Guerrero-Plata A, Rico G, Torres-Guerrero H. Biosynthesis and functions of melanin in *Sporothrix schenckii*. Infect Immun. 2000;68: 3696.

10. Choi Y-E, Goodwin SB. MVE1, Encoding the Velvet Gene Product Homolog in *Mycosphaerella graminicola*, Is Associated with Aerial Mycelium Formation, Melanin Biosynthesis, Hyphal Swelling, and Light Signaling. Appl Environ Microbiol. 2011;77: 942–953. doi:10.1128/AEM.01830-10

11. Choi Y-E, Goodwin SB. Gene Encoding a c-Type Cyclin in *Mycosphaerella graminicola* Is Involved in Aerial Mycelium Formation, Filamentous Growth, Hyphal Swelling, Melanin Biosynthesis, Stress Response, and Pathogenicity. Mol Plant Microbe Interact. 2011;24: 469–477. doi:10.1094/MPMI-04-10-0090

12. Stierle AA, Upadhyay R, Hershenhorn J, Strobel GA, Molina G. The phytotoxins of *Mycosphaerella fijiensis*, the causative agent of Black Sigatoka disease of bananas and plantains. Cell Mol Life Sci CMLS. 1991;47: 853–859.

13. Hoss R, Helbig J, Bochow H. Function of Host and Fungal Metabolites in Resistance Response of Banana and Plantain in the Black Sigatoka Disease Pathosystem (*Musa* spp. *Mycosphaerella fijiensis*). J Phytopathol. 2000;148: 387–394. doi:10.1046/j.1439-0434.2000.00530.x

14. Busogoro JP, Etamé JJ, Lognay G, Messiaen J, Cutsem P van, Lepoivre P. Analysis of the mechanisms of action of *Mycosphaerella fijiensis* toxins during the development of Black Leaf Streak disease. Science Publishers, Inc.; 2004. pp. 171–181.

15. Donzelli BGG, Churchill ACL. A Quantitative Assay Using Mycelial Fragments to Assess Virulence of *Mycosphaerella fijiensis*. Phytopathology. 2007;97: 916–929. doi:10.1094/PHYTO-97-8-0916
