# Supplementary figures and images for: Combating a Global Threat to a Clonal Crop: Banana Black Sigatoka Pathogen Pseudocercospora fijiensis (Synonym Mycosphaerella fijiensis) Genomes Reveal Clues for Disease Control
Source: PLoS Genet. 2016 Aug 11;12(8):e1005876. doi: 10.1371/journal.pgen.1005876 (PMC4981457; doi:10.1371/journal.pgen.1005876)

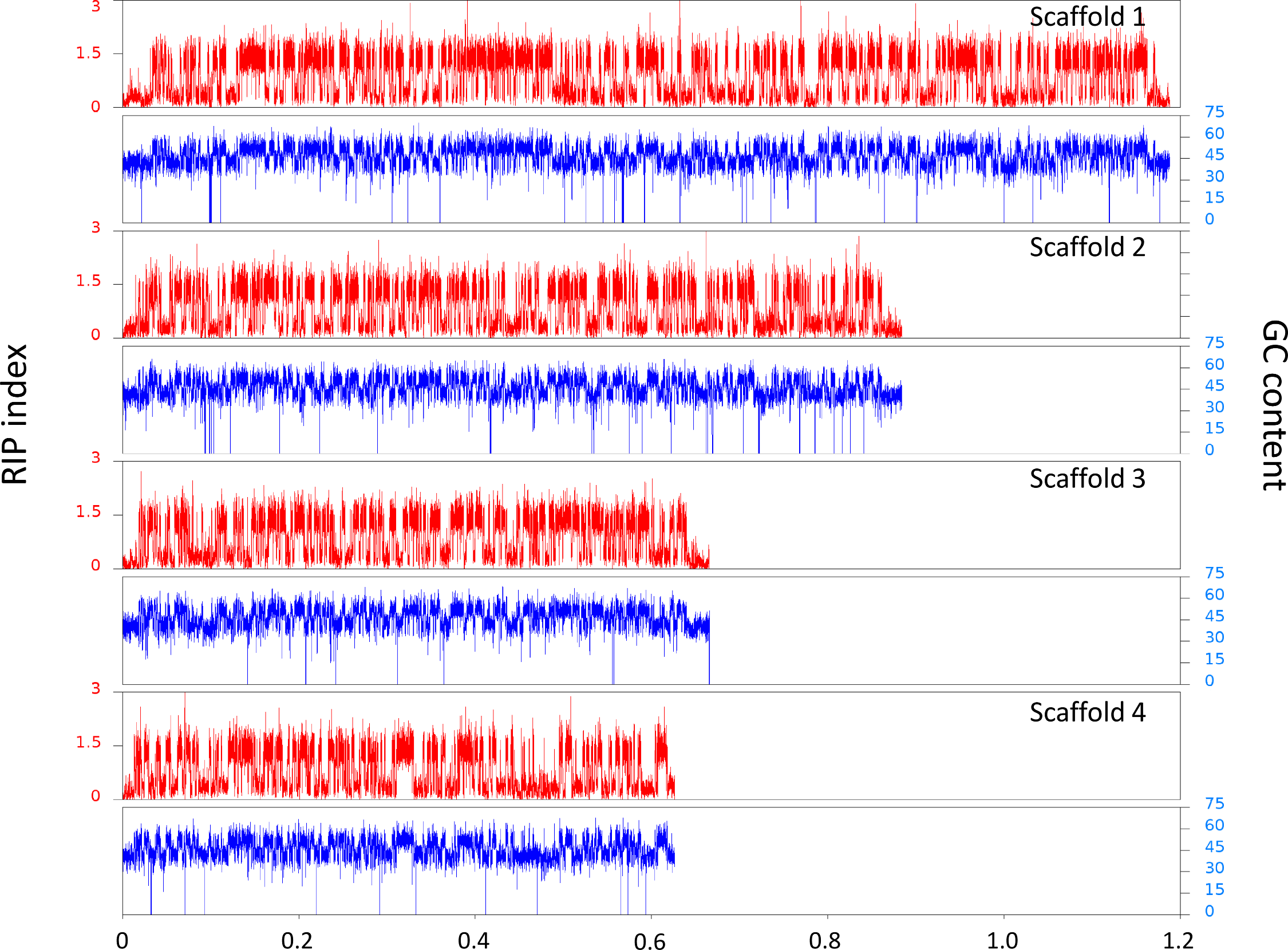

Supplement: S1 Fig — (TIF) [file pgen.1005876.s003.tif]

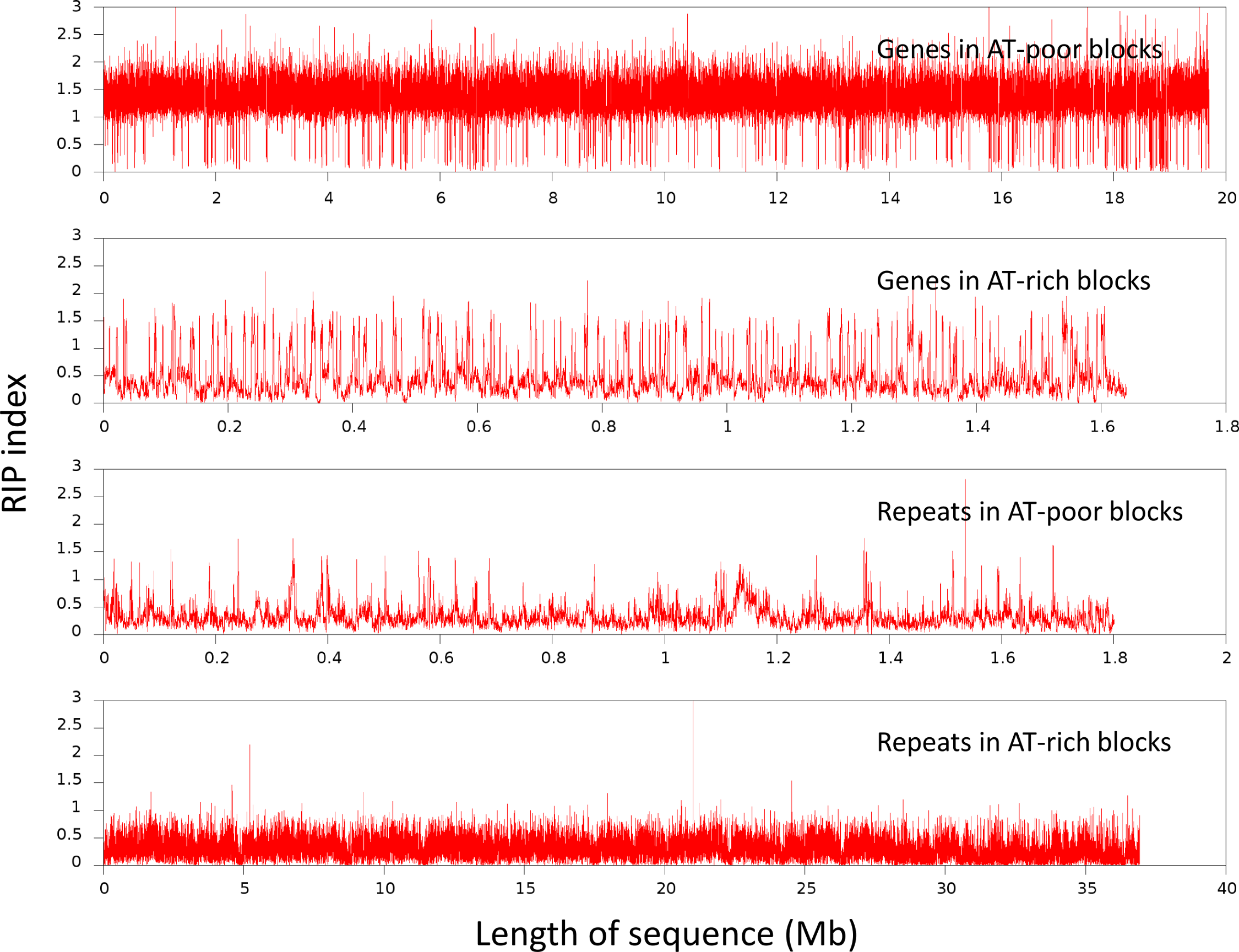

Supplement: S2 Fig — RIP is mostly absent from the genes but highly prevalent among the repeated elements of the genome. (TIF) [file pgen.1005876.s004.tif]

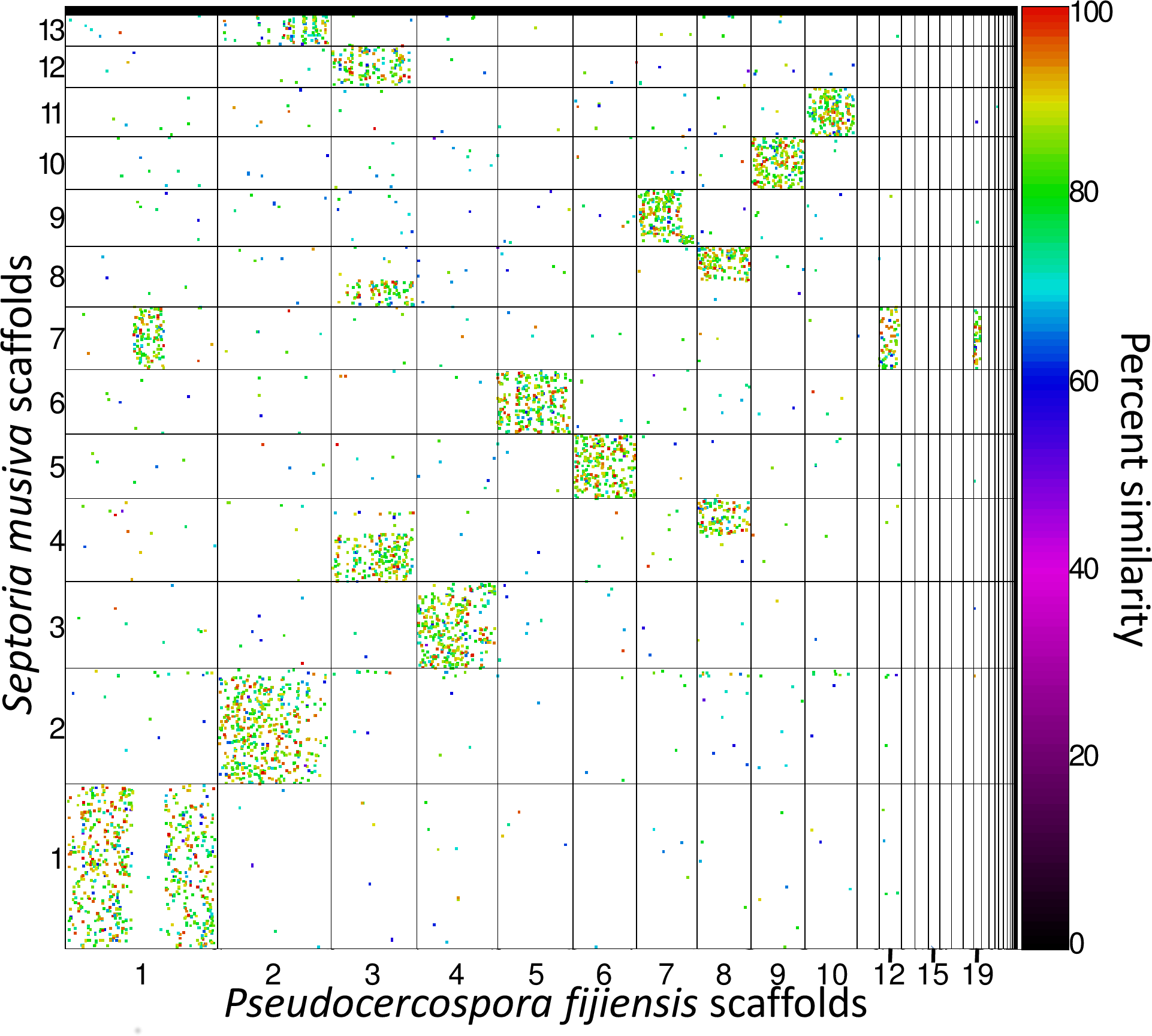

Supplement: S4 Fig — (TIF) [file pgen.1005876.s006.tif]

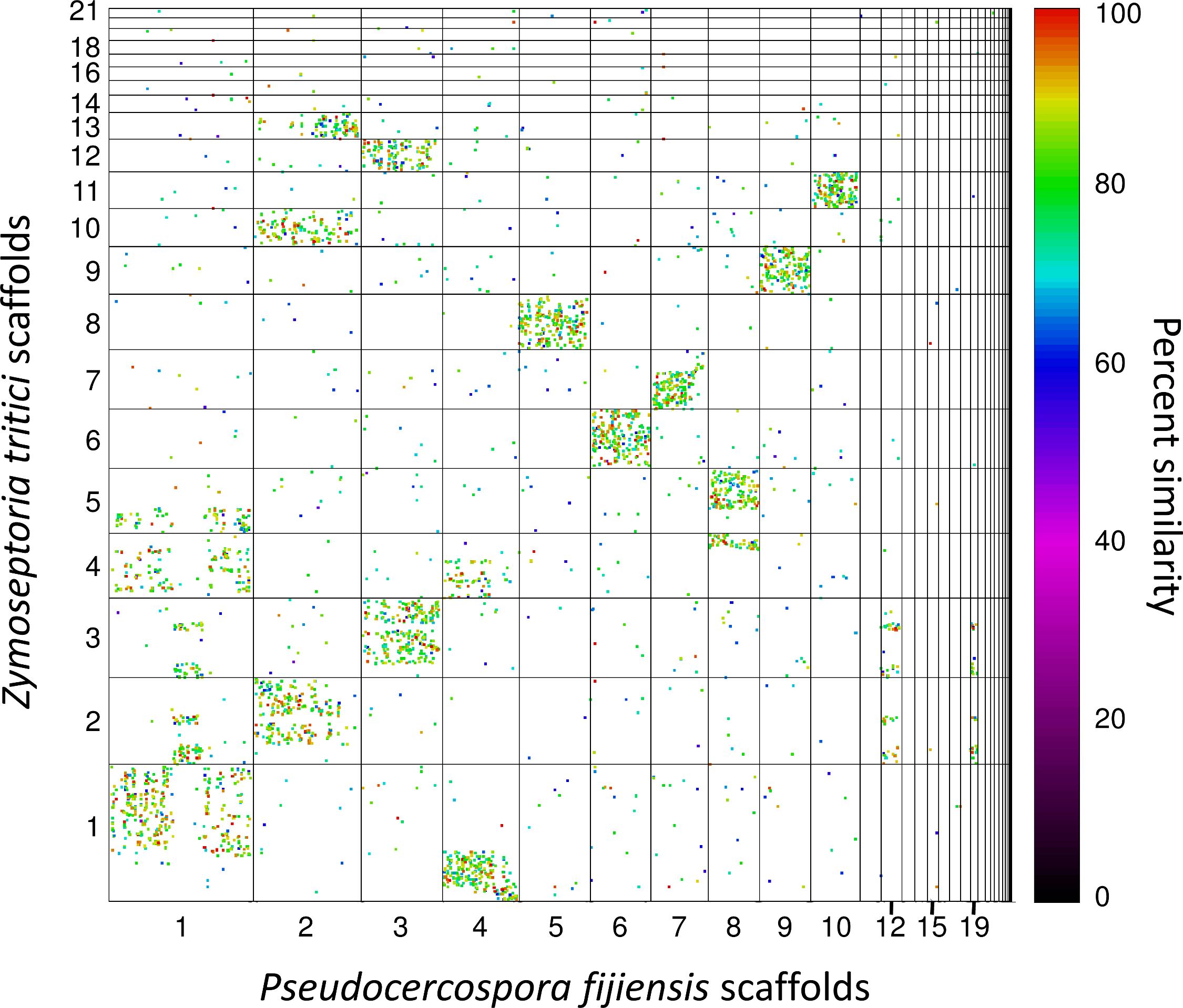

Supplement: S5 Fig — (TIF) [file pgen.1005876.s007.tif]

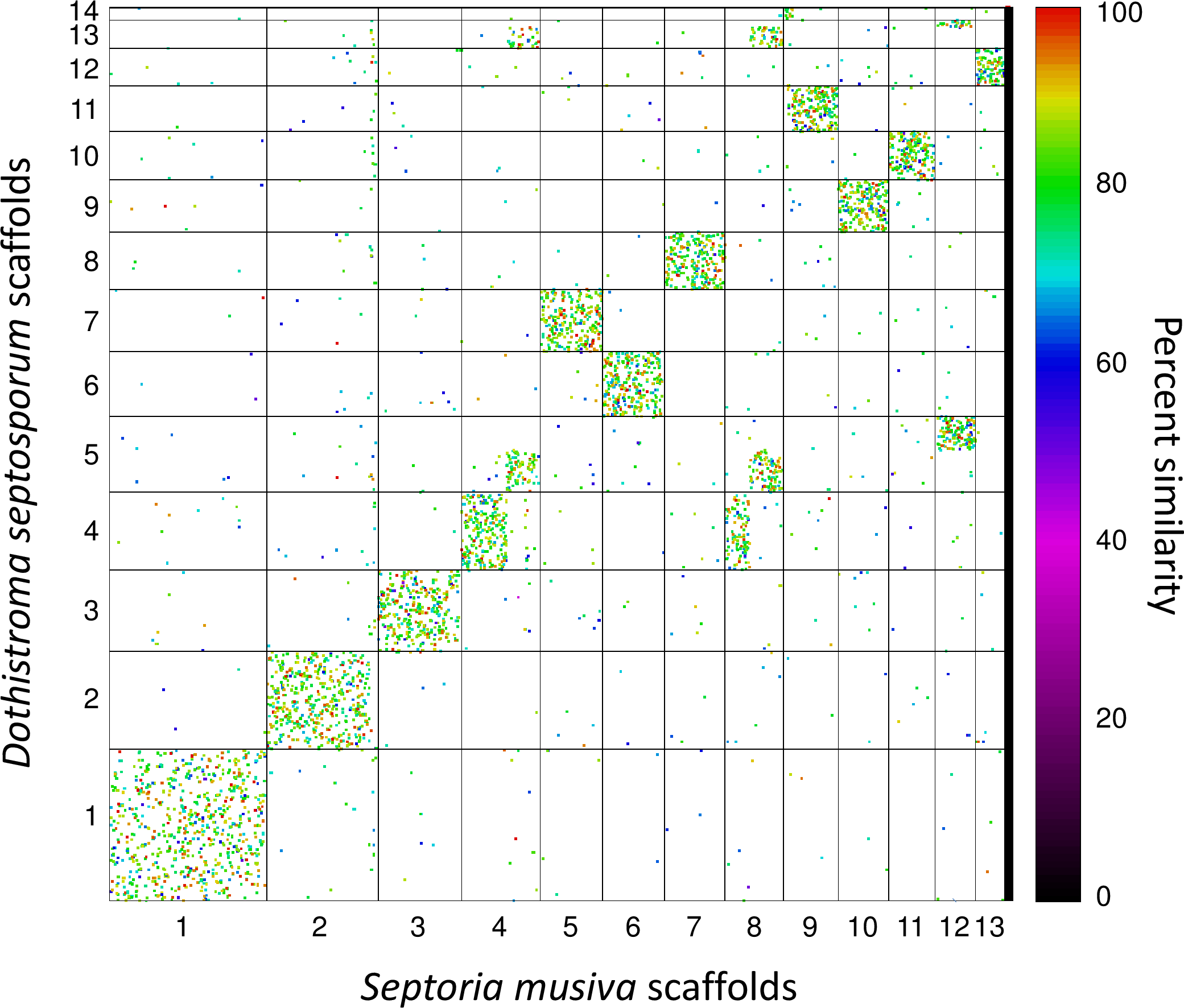

Supplement: S6 Fig — (TIF) [file pgen.1005876.s008.tif]

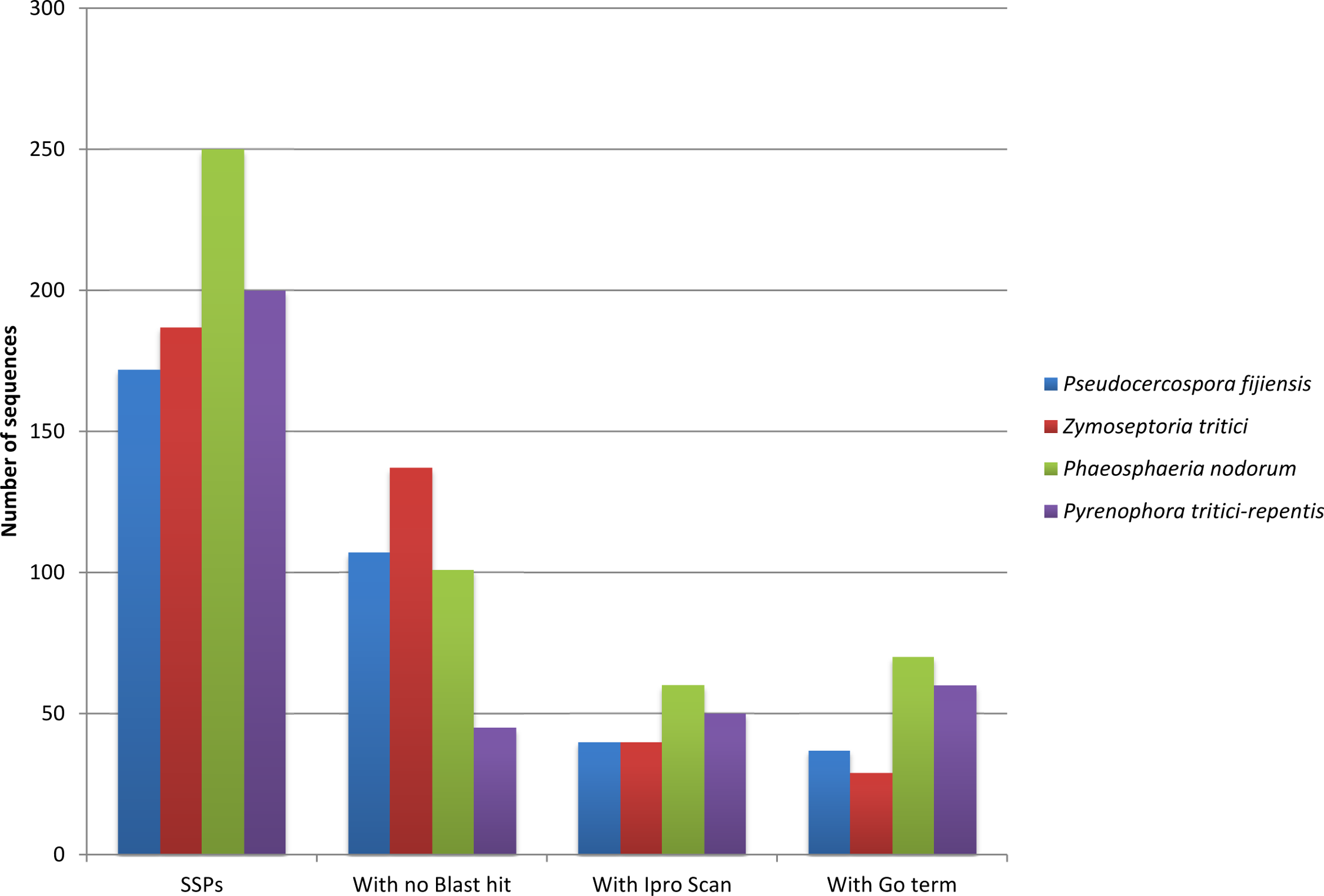

Supplement: S7 Fig — (TIF) [file pgen.1005876.s009.tif]

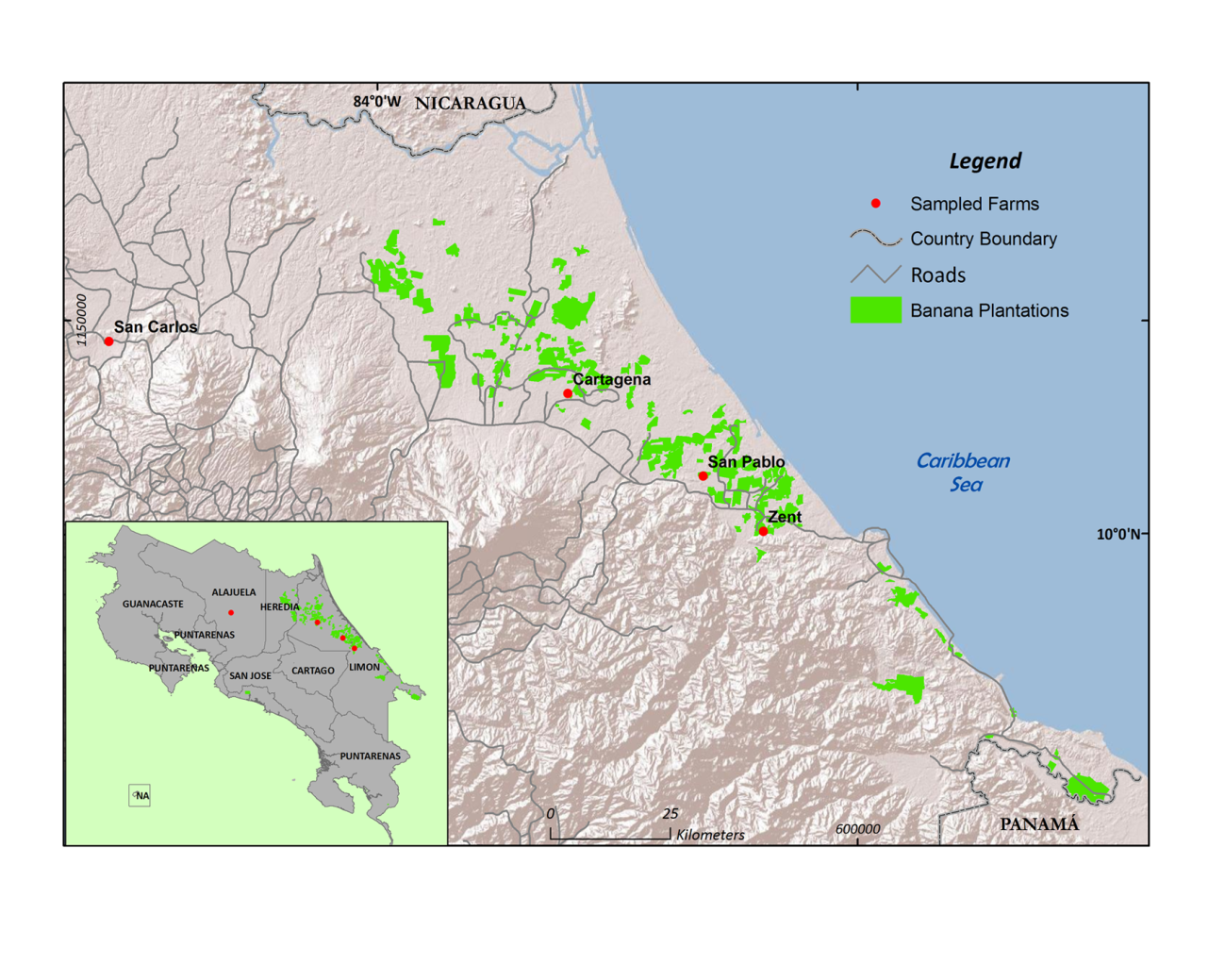

Supplement: S8 Fig — Farms Cartagena, San Pablo and Zent are in a major banana-production area and are sprayed heavily with fungicides; the San Carlos farm is in an area of plantain production (mostly resistant to P. fijiensis) and is not sprayed with fungicides. (DOCX) [file pgen.1005876.s010.docx]
