# Supplementary material for: Combating a Global Threat to a Clonal Crop: Banana Black Sigatoka Pathogen Pseudocercospora fijiensis (Synonym Mycosphaerella fijiensis) Genomes Reveal Clues for Disease Control
Source: PLoS Genet. 2016 Aug 11;12(8):e1005876. doi: 10.1371/journal.pgen.1005876 (PMC4981457; doi:10.1371/journal.pgen.1005876)
Supplement: S1 Table — (DOCX) [file pgen.1005876.s011.docx]

| Scaffold | Size (bp) | G+C, % | Genes | Genes/Mb | Genes, % | Genes with PFAM, % | No. of ESTs | ESTs, % | Repeats, % |
| --- | --- | --- | --- | --- | --- | --- | --- | --- | --- |
| 1 | 11880248 | 46.8 | 2676 | 226.8 | 20.4 | 54.7 | 7277 | 23.00 | 32.6 |
| 2 | 8841024 | 46.2 | 1881 | 214.4 | 14.4 | 52.2 | 4555 | 14.40 | 33.7 |
| 3 | 6657679 | 46.5 | 1526 | 229.8 | 11.6 | 51.0 | 3437 | 10.86 | 31.2 |
| 4 | 6264405 | 45.8 | 1207 | 193.3 | 9.2 | 47.1 | 2725 | 8.61 | 37.0 |
| 5 | 5901819 | 44.8 | 975 | 166.0 | 6.7 | 49.6 | 2149 | 6.79 | 45.2 |
| 6 | 4991523 | 45.0 | 863 | 174.0 | 6.6 | 49.0 | 2413 | 7.63 | 41.7 |
| 7 | 4695110 | 45.7 | 854 | 182.3 | 6.5 | 44.3 | 1953 | 6.17 | 37.6 |
| 8 | 4236865 | 46.2 | 886 | 210.0 | 6.8 | 54.6 | 2030 | 6.42 | 37.4 |
| 9 | 4185348 | 45.2 | 741 | 178.1 | 5.7 | 52.4 | 1297 | 4.10 | 40.1 |
| 10 | 4009308 | 45.4 | 703 | 177.1 | 5.4 | 45.5 | 2842 | 8.98 | 42.1 |
| 11* | 1762310 | 40.2 | 62 | 35.3 | 0.5 | 3.2 | 13 | 0.04 | 66.0 |
| 12 | 1674337 | 44.2 | 253 | 151.4 | 1.9 | 47.0 | 539 | 1.70 | 43.6 |
| 13* | 1121713 | 41.0 | 51 | 45.5 | 0.4 | 0.0 |  |  | 63.1 |
| 14* | 1006785 | 39.7 | 37 | 36.9 | 0.3 | 0.0 | 6 | 0.02 | 60.1 |
| 15* | 928877 | 41.6 | 42 | 45.4 | 0.3 | 4.8 | 13 | 0.04 | 60.8 |
| 16* | 905553 | 39.1 | 2 | 2.2 | 0.0 | 0.0 |  |  | 76.0 |
| 17* | 851953 | 42.1 | 65 | 77.5 | 0.5 | 4.6 | 30 | 0.09 | 32.0 |
| 18* | 830772 | 41.7 | 49 | 59.2 | 0.4 | 2.0 | 38 | 0.12 | 51.5 |
| 19 | 609425 | 46.2 | 130 | 213.8 | 1.0 | 45.4 | 255 | 0.81 | 41.4 |
| 20* | 594030 | 41.1 | 29 | 49.1 | 0.2 | 0.0 | 22 | 0.07 | 41.9 |
| 21* | 427043 | 43.6 | 40 | 94.1 | 0.3 | 2.5 | 21 | 0.07 | 49.4 |
| 22* | 366261 | 41.7 | 30 | 82.3 | 0.2 | 3.3 |  |  | 53.1 |
| 23* | 339557 | 37.5 | 0 | 0.0 | 0.0 | 0.0 |  |  | 66.8 |
| 24* | 325668 | 38.1 | 0 | 0.0 | 0.0 | 0.0 |  |  | 55.2 |
| 25* | 230844 | 42.1 | 1 | 4.4 | 0.0 | 0.0 |  |  | 12.5 |

*Potential dispensable chromosomes are characterized by lower gene densities and higher proportions of repetitive sequences.
