# Supplementary material for: Combating a Global Threat to a Clonal Crop: Banana Black Sigatoka Pathogen Pseudocercospora fijiensis (Synonym Mycosphaerella fijiensis) Genomes Reveal Clues for Disease Control
Source: PLoS Genet. 2016 Aug 11;12(8):e1005876. doi: 10.1371/journal.pgen.1005876 (PMC4981457; doi:10.1371/journal.pgen.1005876)
Supplement: S2 Table — (DOCX) [file pgen.1005876.s012.docx]

| Annotation parameter | v1.0 Assembly | v2.0 Assembly |
| --- | --- | --- |
| Number of gene models | 10,316 | 13,107 |
| Gene density per Mb DNA | 140.5 | 187.6 |
| Average gene length | 1629 nt | 1833 nt |
| Average protein length | 436 aa | 427 aa |
| Average exon frequency ^a^ | 2.45 | 3.62 |
| Average exon length | 578 nt | 395 nt |
| Average intron length | 148 nt | 154 nt |
| Percent complete gene models^b^ | 82 | 88 |
| Percent of genes with homology support | 70 | 74 |
| Percent of genes with Pfam domains | 48 | 49 |
| Percent of genes with EST support |  | 30 |

^a^ Number of exons per gene

^b^ With start and stop codons.
