# Supplementary material for: Combating a Global Threat to a Clonal Crop: Banana Black Sigatoka Pathogen Pseudocercospora fijiensis (Synonym Mycosphaerella fijiensis) Genomes Reveal Clues for Disease Control
Source: PLoS Genet. 2016 Aug 11;12(8):e1005876. doi: 10.1371/journal.pgen.1005876 (PMC4981457; doi:10.1371/journal.pgen.1005876)
Supplement: S3 Table — (DOCX) [file pgen.1005876.s013.docx]

|  |  | Percent G+C | | Number of |
| --- | --- | --- | --- | --- |
| Species | Isolate | Peak 1 | Peak 2 | replications |
| *P. fijiensis* | CIRAD86 | 39.4 ± 0.80 | 51.6 ± 0.90 | 18 |
| *P. eumusae* | CBS122457 | 39.6 ± 0.03 | 51.6 ± 0.08 | 2 |
| *P. musae* | UQ430 | 37.2 ± 1.30 | 50.9 ± 0.50 | 6 |
| *Z. tritici* | IPO323 | — | 53.1 ± 0.50 | 6 |
