# Supplementary material for: Combating a Global Threat to a Clonal Crop: Banana Black Sigatoka Pathogen Pseudocercospora fijiensis (Synonym Mycosphaerella fijiensis) Genomes Reveal Clues for Disease Control
Source: PLoS Genet. 2016 Aug 11;12(8):e1005876. doi: 10.1371/journal.pgen.1005876 (PMC4981457; doi:10.1371/journal.pgen.1005876)
Supplement: S4 Table — (DOCX) [file pgen.1005876.s014.docx]

| Parameter | Number (percent) |
| --- | --- |
| Total reads | 73,185,656 |
| Uniquely aligned | 43,622,808 (59.6%) |
| Ambiguously mapped | 20,982,018 (28.7%) |
| Unmapped | 8,580,830 (11.7%) |
| Genome coverage (bp) | 65,956,770 (89%) |
| Average depth of coverage | 35.6 x |
| Total variants | 514,953 |
| Total SNPs | 509,749 (99%) |
| Total indels | 5,204 (1%) |
| Variants in coding sequences | 95,335 (18.5%) |
| Non-synonymous variants | 46,295 (9%) |
