# Supplementary material for: Combating a Global Threat to a Clonal Crop: Banana Black Sigatoka Pathogen Pseudocercospora fijiensis (Synonym Mycosphaerella fijiensis) Genomes Reveal Clues for Disease Control
Source: PLoS Genet. 2016 Aug 11;12(8):e1005876. doi: 10.1371/journal.pgen.1005876 (PMC4981457; doi:10.1371/journal.pgen.1005876)
Supplement: S5 Table — (DOCX) [file pgen.1005876.s015.docx]

| Scaffold | Length  (10^4^ bp) | Total variants | Substitutions | Indels | Mean frequency  (per 10kb) |
| --- | --- | --- | --- | --- | --- |
| 1 | 1188 | 71940 | 70994 | 946 | 60.6 |
| 2 | 885 | 58469 | 57793 | 676 | 66.1 |
| 3 | 667 | 44949 | 44352 | 597 | 67.4 |
| 4 | 627 | 45844 | 45299 | 545 | 73.1 |
| 5 | 591 | 41410 | 41033 | 377 | 70.1 |
| 6 | 500 | 37467 | 37088 | 379 | 74.9 |
| 7 | 470 | 36909 | 36542 | 367 | 78.5 |
| 8 | 425 | 28845 | 28473 | 372 | 67.9 |
| 9 | 420 | 28315 | 28021 | 294 | 67.4 |
| 10 | 402 | 28985 | 28707 | 278 | 72.1 |
| 11 | 177 | 14887 | 14833 | 54 | 84.1 |
| 12 | 168 | 12317 | 12204 | 113 | 73.3 |
| 13 | 113 | 6695 | 6691 | 4 | 59.2 |
| 14 | 102 | 8049 | 8027 | 22 | 78.9 |
| 15 | 94 | 6700 | 6668 | 32 | 71.3 |
| 16 | 91 | 6292 | 6277 | 15 | 69.1 |
| 17 | 86 | 6301 | 6281 | 20 | 73.3 |
| 18 | 84 | 6155 | 6130 | 25 | 73.3 |
| 19 | 62 | 3781 | 3743 | 38 | 61.0 |
| 20 | 60 | 4235 | 4234 | 1 | 70.6 |
| 21 | 44 | 3517 | 3492 | 25 | 79.9 |
