# Supplementary material for: Combating a Global Threat to a Clonal Crop: Banana Black Sigatoka Pathogen Pseudocercospora fijiensis (Synonym Mycosphaerella fijiensis) Genomes Reveal Clues for Disease Control
Source: PLoS Genet. 2016 Aug 11;12(8):e1005876. doi: 10.1371/journal.pgen.1005876 (PMC4981457; doi:10.1371/journal.pgen.1005876)
Supplement: S6 Table — (DOCX) [file pgen.1005876.s016.docx]

|  |  | Fungicide sensitivity | | |  | Mating type | | |
| --- | --- | --- | --- | --- | --- | --- | --- | --- |
| Population | Sample size^a^ | Sensitive | Resistant | Percent resistant |  | Ratio | χ^2^ | *P*^b^ |
| Cartagena | 189 | 0 | 178 | 100.0 |  | 90:85 | 0.14 | 0.705 |
| San Carlos | 91 | 87 | 0 | 0.0 |  | 42:45 | 0.10 | 0.748 |
| San Pablo | 179 | 0 | 180 | 100.0 |  | 84:82 | 0.02 | 0.877 |
| Zent | 190 | 14 | 162 | 92.0 |  | 77:96 | 2.09 | 0.149 |
| Total or mean | 649 | 101 | 520 | 83.7 |  | 293:308 | 0.37 | 0.541 |

^a^ Total number of isolates with molecular marker data for at least one locus. Sample sizes for each statistic varied depending on the number of isolates scored successfully.

^b^ Probability that the observed mating type ratios are not significantly different from 1:1.
