# Supplementary material for: Combating a Global Threat to a Clonal Crop: Banana Black Sigatoka Pathogen Pseudocercospora fijiensis (Synonym Mycosphaerella fijiensis) Genomes Reveal Clues for Disease Control
Source: PLoS Genet. 2016 Aug 11;12(8):e1005876. doi: 10.1371/journal.pgen.1005876 (PMC4981457; doi:10.1371/journal.pgen.1005876)
Supplement: S7 Table — (DOCX) [file pgen.1005876.s017.docx]

| Population | Cartagena | San Carlos | San Pablo | Zent |
| --- | --- | --- | --- | --- |
| Cartagena | —^c^ | 0.067 | 0.052 | 0.089 |
| San Carlos | 0.067 | — | 0.192 | 0.126 |
| San Pablo | 0.116 | 0.097 | — | 0.065 |
| Zent | 0.190 | 0.243 | 0.146 | — |

^a^ *D* and *G*′′*_ST_* were calculated with GenoDive (Meirmans and van Tienderen, 2004) as described by Jost (2008) and Meirmans and Hedrick (2011), respectively.

^b^ All values for each statistic were significantly different at *P* = 0.001.

^c^ Not applicable to self comparisons.
