# Supplementary material for: Combating a Global Threat to a Clonal Crop: Banana Black Sigatoka Pathogen Pseudocercospora fijiensis (Synonym Mycosphaerella fijiensis) Genomes Reveal Clues for Disease Control
Source: PLoS Genet. 2016 Aug 11;12(8):e1005876. doi: 10.1371/journal.pgen.1005876 (PMC4981457; doi:10.1371/journal.pgen.1005876)
Supplement: S8 Table — (DOCX) [file pgen.1005876.s018.docx]

| Annotation parameter | Number or percent of genes |
| --- | --- |
| Number of gene models | 13,107 |
| Percent complete (with start and stop codons) | 88% |
| Percent of genes with homology support | 74% |
| Percent of genes with Pfam domains | 49% |
| Percent of genes with EST support | 30% |
