# Supplementary material for: Combating a Global Threat to a Clonal Crop: Banana Black Sigatoka Pathogen Pseudocercospora fijiensis (Synonym Mycosphaerella fijiensis) Genomes Reveal Clues for Disease Control
Source: PLoS Genet. 2016 Aug 11;12(8):e1005876. doi: 10.1371/journal.pgen.1005876 (PMC4981457; doi:10.1371/journal.pgen.1005876)
Supplement: S9 Table — (DOCX) [file pgen.1005876.s019.docx]

| Category | Number of genes (Percent) |
| --- | --- |
| Proteins assigned to a KOG | 6676 (51%) |
| KOG categories genome-wide | 3247 |
| Proteins assigned a GO term | 5655 (43%) |
| GO terms genome-wide | 1393 |
| Proteins assigned an EC number | 2666 (20%) |
| EC numbers genome-wide | 798 |
| Proteins assigned a Pfam domain | 6603 (50%) |
| Pfam domains genome wide | 2446 |
