# Supplementary material for: Combating a Global Threat to a Clonal Crop: Banana Black Sigatoka Pathogen Pseudocercospora fijiensis (Synonym Mycosphaerella fijiensis) Genomes Reveal Clues for Disease Control
Source: PLoS Genet. 2016 Aug 11;12(8):e1005876. doi: 10.1371/journal.pgen.1005876 (PMC4981457; doi:10.1371/journal.pgen.1005876)
Supplement: S10 Table — (DOCX) [file pgen.1005876.s020.docx]

| Locus | Forward primer^a^ | Reverse primer^a^ | Expected size |
| --- | --- | --- | --- |
| Fungicide sensitivity | |  |  |
| *cytb* | CTCAAATACTGCCTCAGC | CCGTAATGTGGTTCATC | 285 |
|  | CTCAAATACTGCCTCAGC | GTTATAACTGTAGCTCC | 198 |
| Variable Number of Tandem repeats (VNTR) loci | | | |
| 1333 | GAGTGAAGTACTGCGGAGGC | AGTTGGAGAAAGGCGAAAGG | 259 |
| 3959 | GCGCGAGGCTTTCTATCTC | ACCCCGATTAGGGAAGGTC | 184 |
| 3786 | GCAGCGGAGTGCTAGTAACC | CGCGCTTTTGACTCTTCTTC | 260 |
| 0252 | TAGAGGCTACCCTGCCGTC | GTATACTTCCGACCTCGGGC | 132 |
| 0705 | ATAGGATGCGGCAGACACTC | CGTCGCGATTTGAAGTGCC | 214 |

^a^ Primer sequences written 5’ to 3’.
